# Supplementary material for: Enhancing microalgal productivity through bioactive substances, light, and CO2
Source: PLoS One. 2026 Apr 27;21(4):e0338585. doi: 10.1371/journal.pone.0338585 (PMC13119868; doi:10.1371/journal.pone.0338585)
Supplement: S1 File — (DOCX) [file pone.0338585.s001.docx]

**A.** Effect of 10 bioactive substances with 4 concentrations, 4 wavelengths, and 4 CO_2_ injection times on the growth of *A. platensis*.

| **Bioactive substances substances** | | | | |
| --- | --- | --- | --- | --- |
| Control | 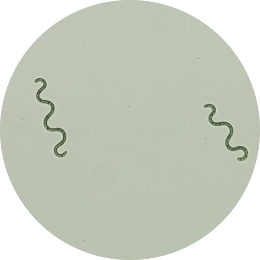 | | | |
| N-Butyryl-DL-homoserine lactone | 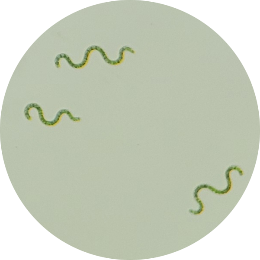 | 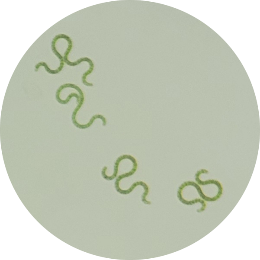 | 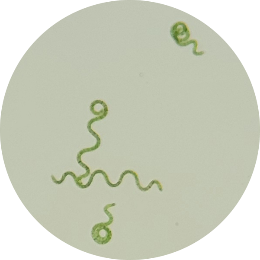 | 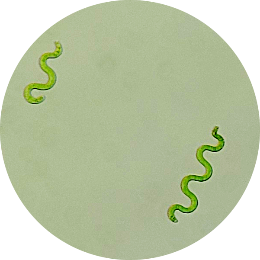 |
|  | C1=0.1μg/mL | C2=1.0μg/mL | C3=5.0μg/mL | C4=10.0μg/mL |
| L-Homoserine lactone hydrochloride | 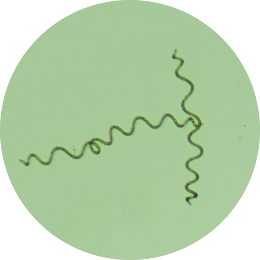 | 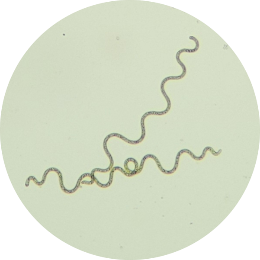 | 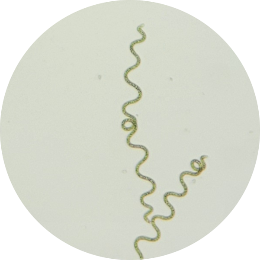 | 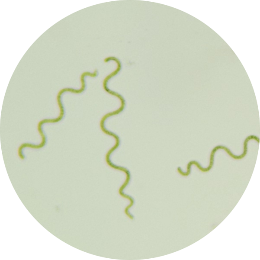 |
|  | C1=0.1μg/mL | C2=1.0μg/mL | C3=5.0μg/mL | C4=10.0μg/mL |
| Indole-3-butyric acid | 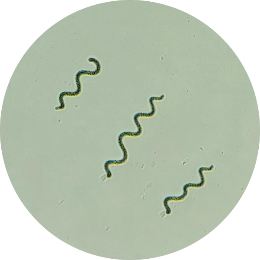 | 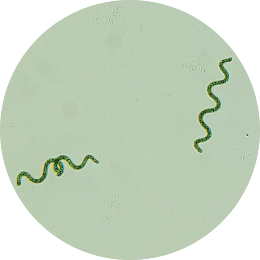 | 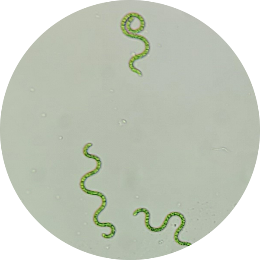 | 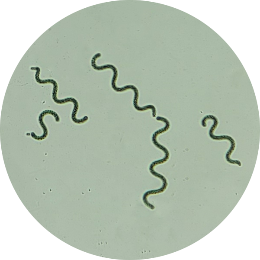 |
|  | C1=0.1μg/mL | C2=1.0μg/mL | C3=5.0μg/mL | C4=10.0μg/mL |
| 1-Naphthaleneacetic aci | 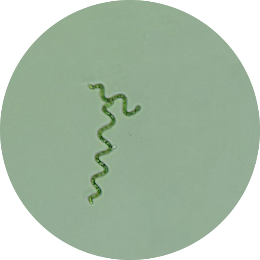 | 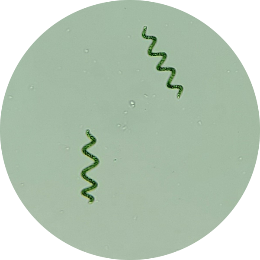 | 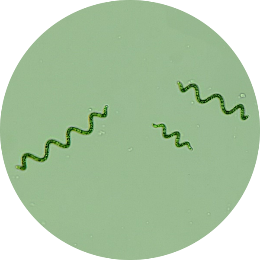 | 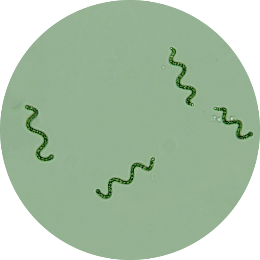 |
|  | C1=0.1μg/mL | C2=1.0μg/mL | C3=5.0μg/mL | C4=10.0μg/mL |
| Indole-3-acetic acid | 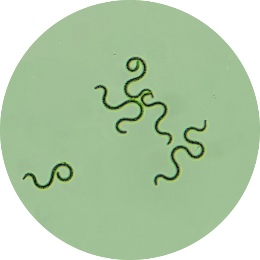 | 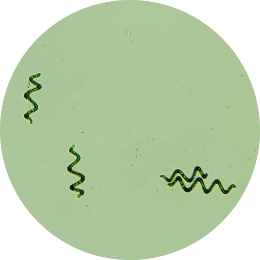 | 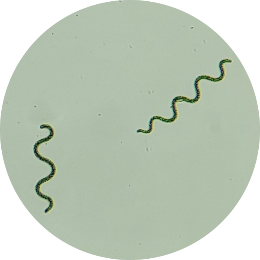 | 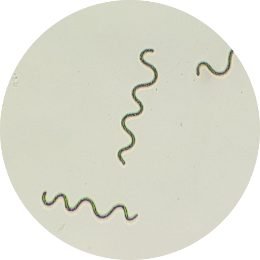 |
|  | C1=0.1μg/mL | C2=1.0μg/mL | C3=5.0μg/mL | C4=10.0μg/mL |
| Salicylic acid | 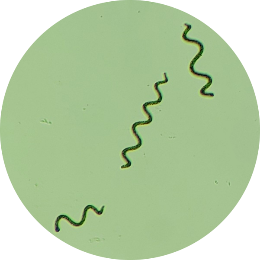 | 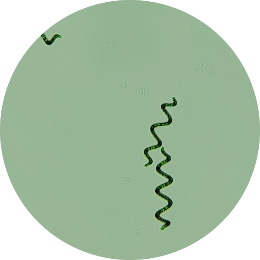 | 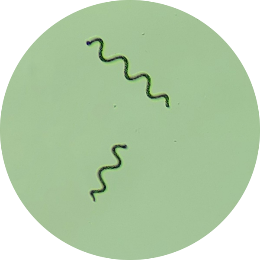 | 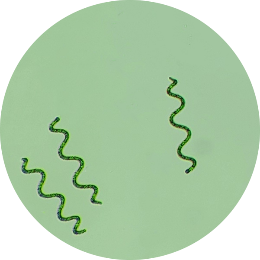 |
|  | C1=0.1μg/mL | C2=1.0μg/mL | C3=5.0μg/mL | C4=10.0μg/mL |
| Coconut water | 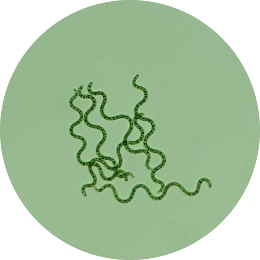 | 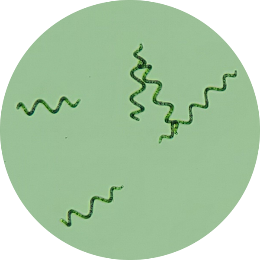 | 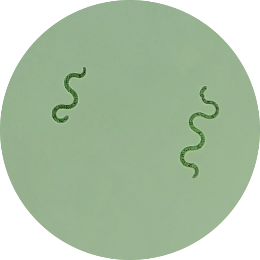 | 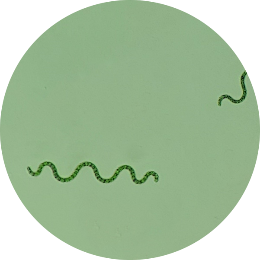 |
|  | C1=1% | C2=3% | C3=7% | C4=10% |
| *Aloe vera* | 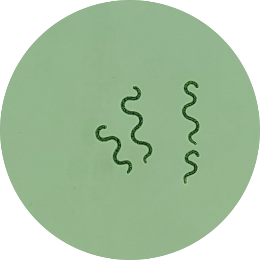 | 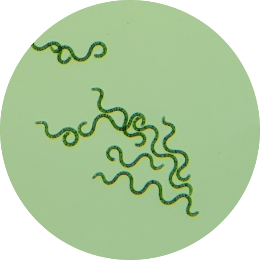 | 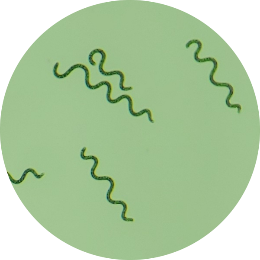 | 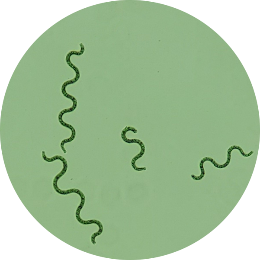 |
|  | C1=1% | C2=3% | C3=7% | C4=10% |
| Lentil sprout extract | 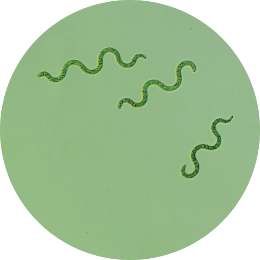 | 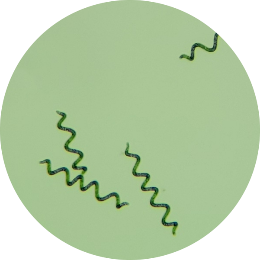 | 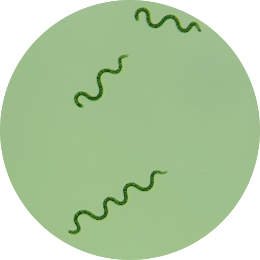 | 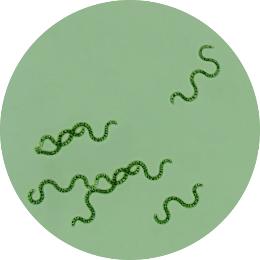 |
|  | C1=0.1μg/mL | C2=1.0μg/mL | C3=5.0μg/mL | C4=10.0μg/mL |
| Sargassum extract | 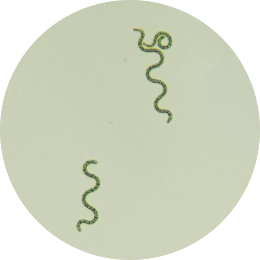 | 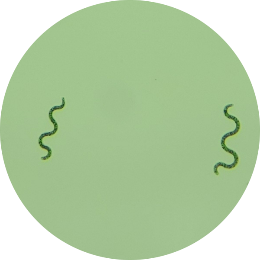 | 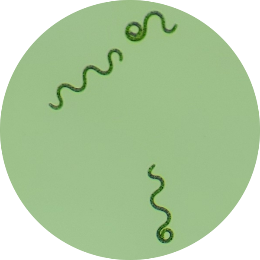 | 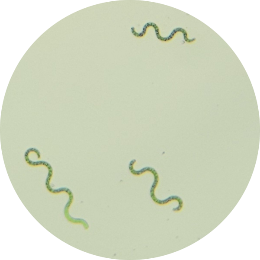 |
|  | C1=0.1μg/mL | C2=1.0μg/mL | C3=5.0μg/mL | C4=10.0μg/mL |
| **Wavelength** | | | | |
| Lights | 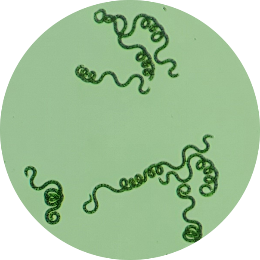 | 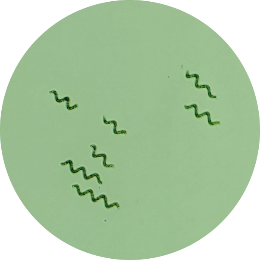 | 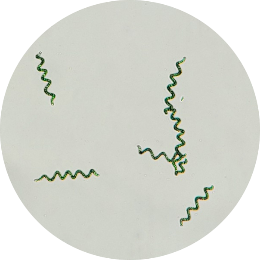 | 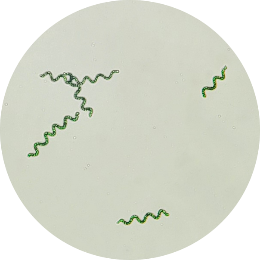 |
|  | L1= Blue light | L2= Red light | L3= Green light | L4= Yellow light |
| **CO_2_** | | | | |
| CO_2_ | 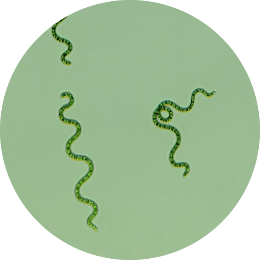 | 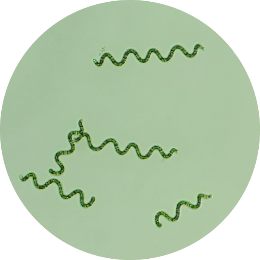 | 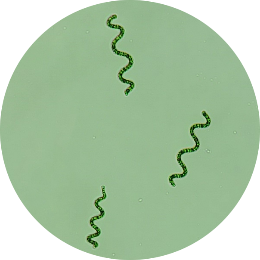 | 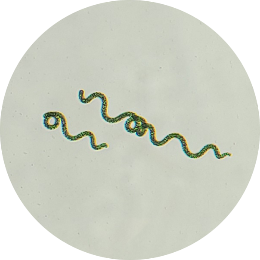 |
|  | T1=30s | T2=60s | T3=90s | T4=120s |

**B.** Effect of 7 culture conditions on the growth of *A. platensis.*

| 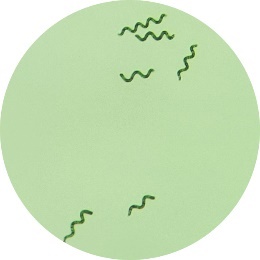 | 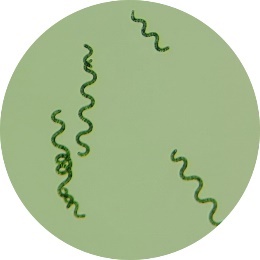 | 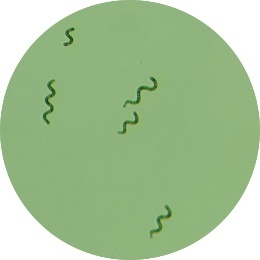 | 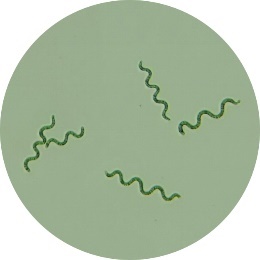 |
| --- | --- | --- | --- |
| Control | C1 - *Aloe vera* 3% | C2 - Red light (600-700nm) | C3 - 60s of CO_2_ |
| 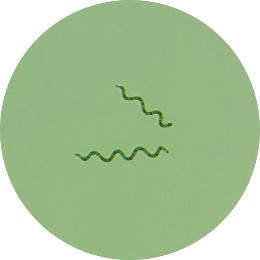 | 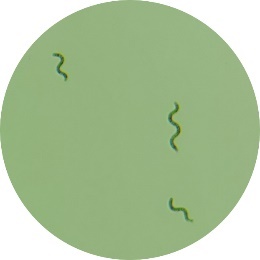 | 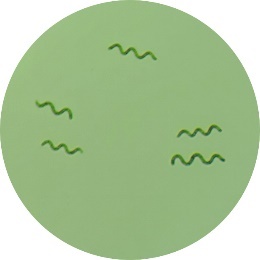 | 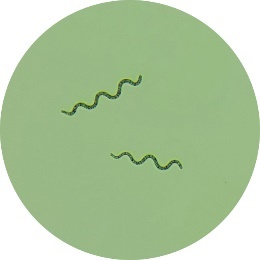 |
| C4 - *Aloe vera* 3% + Red light (600-700nm) + 60s of CO_2_ | C5 - *Aloe vera* 3%+ Red light (600-700nm) | C6 - *Aloe vera* 3% + 60s of CO_2_ | C7 - Red light (600-700nm) + 60s of CO_2_ |

**C.** Effect of 10 bioactive substances with their 4 concentrations, 4 wavelengths, and 4 CO_2_ injection times on the growth of *C. vulgaris*.

| **Bioactive substances** | | | | |
| --- | --- | --- | --- | --- |
| Control | 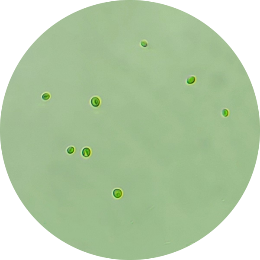 | | | |
| N-Butyryl-DL-homoserine lactone | 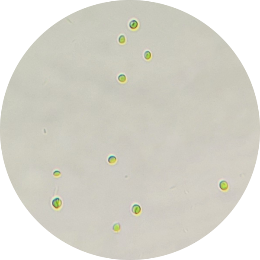 | 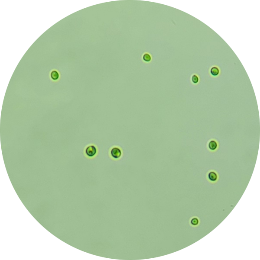 | 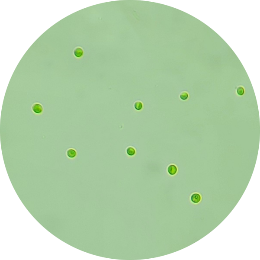 | 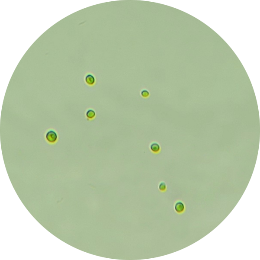 |
|  | C1=0.1μg/mL | C2=1.0μg/mL | C3=5.0μg/mL | C4=10.0μg/mL |
| L-Homoserine lactone hydrochloride | 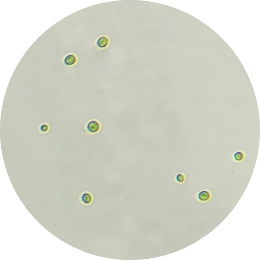 | 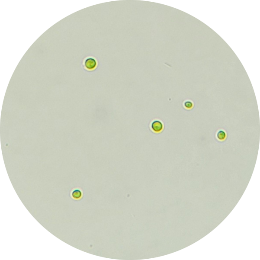 | 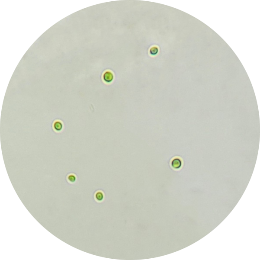 | 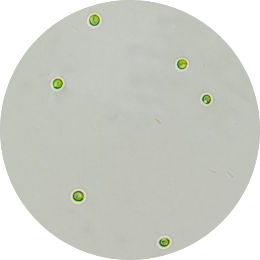 |
|  | C1=0.1μg/mL | C2=1.0μg/mL | C3=5.0μg/mL | C4=10.0μg/mL |
| Indole-3-butyric acid | 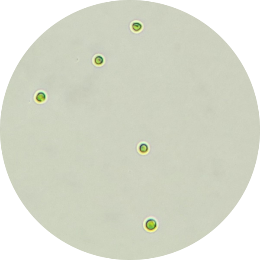 | 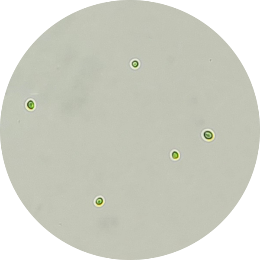 | 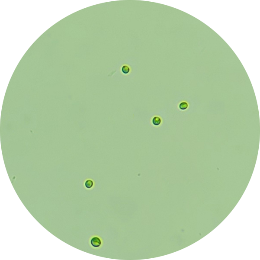 | 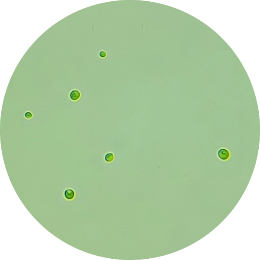 |
|  | C1=0.1μg/mL | C2=1.0μg/mL | C3=5.0μg/mL | C4=10.0μg/mL |
| 1-Naphthaleneacetic acid | 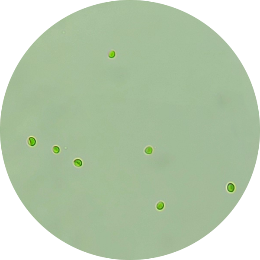 | 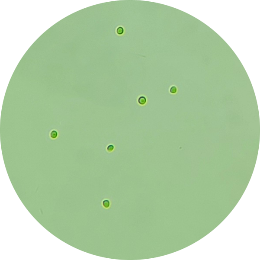 | 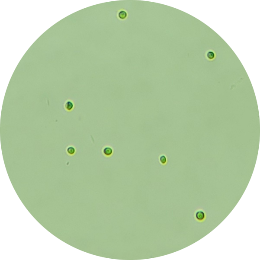 | 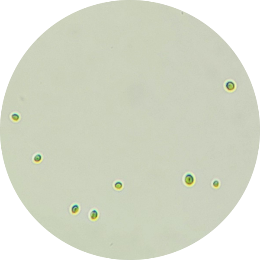 |
|  | C1=0.1μg/mL | C2=1.0μg/mL | C3=5.0μg/mL | C4=10.0μg/mL |
| Indole-3-acetic acid | 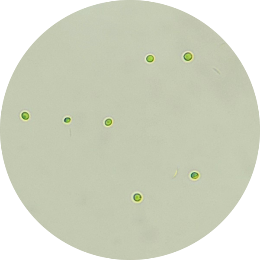 | 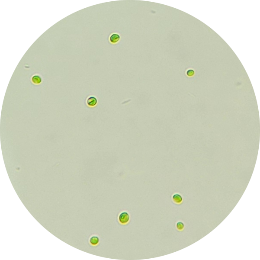 | 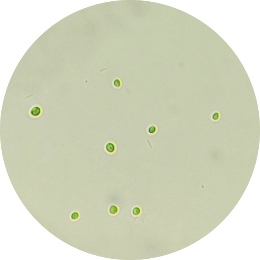 | 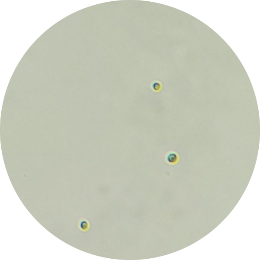 |
|  | C1=0.1μg/mL | C2=1.0μg/mL | C3=5.0μg/mL | C4=10.0μg/mL |
| Salicylic acid | 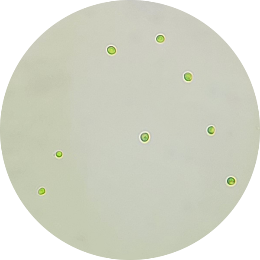 | 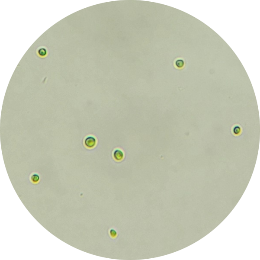 | 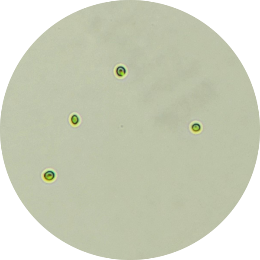 | 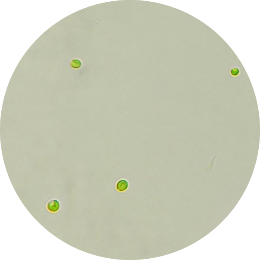 |
|  | C1=0.1μg/mL | C2=1.0μg/mL | C3=5.0μg/mL | C4=10.0μg/mL |
| Coconut water | 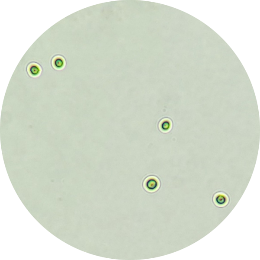 | 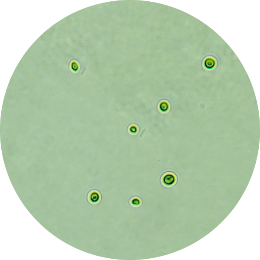 | 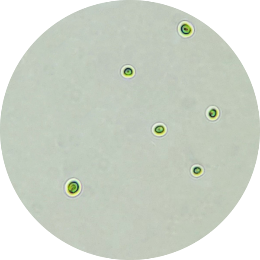 | 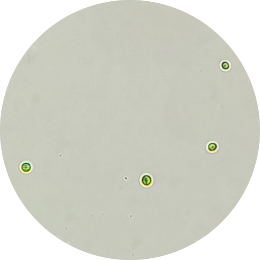 |
|  | C1=1% | C2=3% | C3=7% | C4=10% |
| *Aloe vera* | 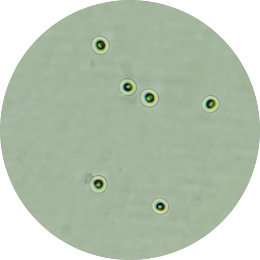 | 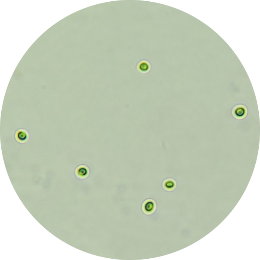 | 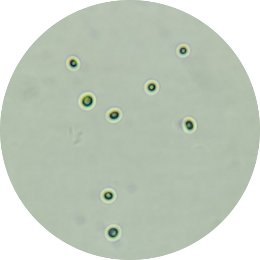 | 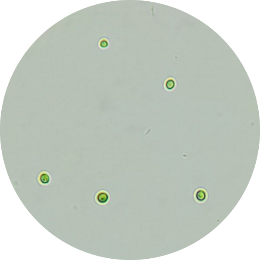 |
|  | C1=1% | C2=3% | C3=7% | C4=10% |
| Lentil sprout extract | 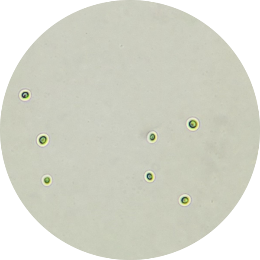 | 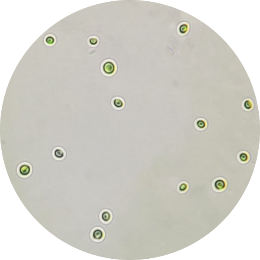 | 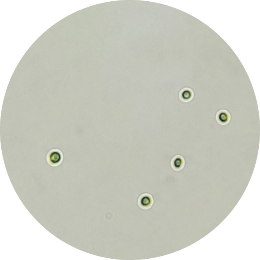 | 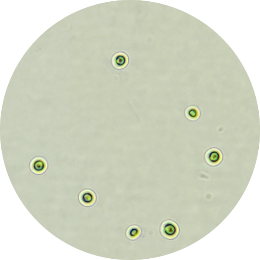 |
|  | C1=0.1μg/mL | C2=1.0μg/mL | C3=5.0μg/mL | C4=10.0μg/mL |
| Sargassum extract | 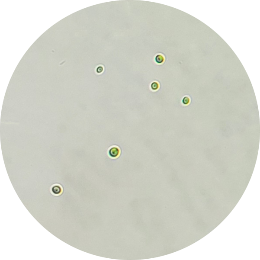 | 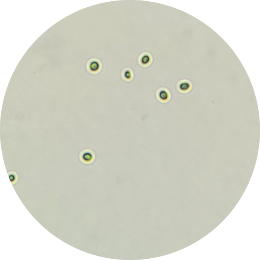 | 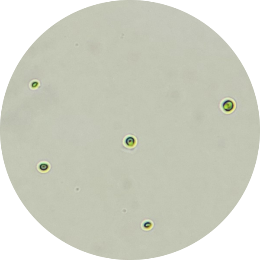 | 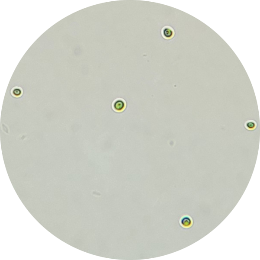 |
|  | C1=0.1μg/mL | C2=1.0μg/mL | C3=5.0μg/mL | C4=10.0μg/mL |
| **Wavelength** | | | | |
| Lights | 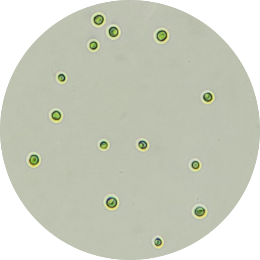 | 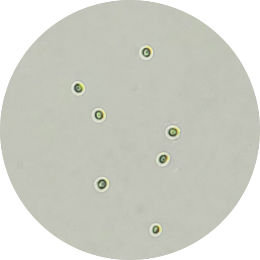 |  |  |
|  | L1= Blue light | L2= Red light | L3= Green light | L4= Yellow light |
| **CO_2_** | | | | |
| CO_2_ |  |  |  |  |
|  | T1=30s | T2=60s | T3=90s | T4=120s |

**D.** Effect of 7 culture conditions on the growth of *C. vulgaris.*

|  |  |  |  |
| --- | --- | --- | --- |
| Control | C1 - *Aloe vera* 3% | C2 - Blue light (400-490nm) | C3 - 60s of CO_2_ |
|  |  |  |  |
| C4 - *Aloe vera* 3% + Blue light (400-490nm) + 60s of CO_2_ | C5 - *Aloe vera* 3% + Blue light (400-490nm) | C6 - *Aloe vera* 3% + 60s of CO_2_ | C7 - Blue light (400-490nm) + 60s of CO_2_ |

**E.** Effect of 10 bioactive substances with their 4 concentrations, 4 wavelengths, and 4 CO_2_ injection times on the growth of *A. falcatus.*

| **Bioactive substances** | | | | |
| --- | --- | --- | --- | --- |
| Control |  | | | |
| N-Butyryl-DL-homoserine lactone |  |  |  |  |
|  | C1=0.1μg/mL | C2=1.0μg/mL | C3=5.0μg/mL | C4=10.0μg/mL |
| L-Homoserine lactone hydrochloride |  |  |  |  |
|  | C1=0.1μg/mL | C2=1.0μg/mL | C3=5.0μg/mL | C4=10.0μg/mL |
| Indole-3-butyric acid |  |  |  |  |
|  | C1=0.1μg/mL | C2=1.0μg/mL | C3=5.0μg/mL | C4=10.0μg/mL |
| 1-Naphthaleneacetic acid |  |  |  |  |
|  | C1=0.1μg/mL | C2=1.0μg/mL | C3=5.0μg/mL | C4=10.0μg/mL |
| Indole-3-acetic acid |  |  |  |  |
|  | C1=0.1μg/mL | C2=1.0μg/mL | C3=5.0μg/mL | C4=10.0μg/mL |
| Salicylic acid |  |  |  |  |
|  | C1=0.1μg/mL | C2=1.0μg/mL | C3=5.0μg/mL | C4=10.0μg/mL |
| Coconut water |  |  |  |  |
|  | C1=1% | C2=3% | C3=7% | C4=10% |
| *Aloe vera* |  |  |  |  |
|  | C1=1% | C2=3% | C3=7% | C4=10% |
| Lentil sprout extract |  |  |  |  |
|  | C1=0.1μg/mL | C2=1.0μg/mL | C3=5.0μg/mL | C4=10.0μg/mL |
| Sargassum extract |  |  |  |  |
|  | C1=0.1μg/mL | C2=1.0μg/mL | C3=5.0μg/mL | C4=10.0μg/mL |
| **Wavelength** | | | | |
| Lights |  |  |  |  |
|  | L1=Blue light | L2=Red light | L3=Green light | L4=Yellow light |
| **CO_2_** | | | | |
| CO_2_ |  |  |  |  |
|  | T1=30s | T2=60s | T3=90s | T4=120s |

**F.** Effect of 7 culture conditions on the growth of *A. falcatus.*

|  |  |  |  |
| --- | --- | --- | --- |
| Control | C1 - *Aloe vera* 1% | C2 - Red light (600-700nm) | C3 - 30s of CO_2_ |
|  |  |  |  |
| C4 - *Aloe vera* 1% + Red light (600-700nm) + 30s of CO_2_ | C5 - *Aloe vera* 1% + Red light (600-700nm) | C6 - *Aloe vera* 1% + 30s of CO_2_ | C7 - Red light (600-700nm) + 30s of CO_2_ |

**G.** Effect of 10 bioactive substances with their 4 concentrations, 4 wavelengths, and 4 CO_2_ injection times on the growth of *T. dimorphus.*

| **Bioactive substances** | | | | |
| --- | --- | --- | --- | --- |
| Control |  | | | |
| N-Butyryl-DL-homoserine lactone |  |  |  |  |
|  | C1=0.1μg/mL | C2=1.0μg/mL | C3=5.0μg/mL | C4=10.0μg/mL |
| L-Homoserine lactone hydrochloride |  |  |  |  |
|  | C1=0.1μg/mL | C2=1.0μg/mL | C3=5.0μg/mL | C4=10.0μg/mL |
| Indole-3-butyric acid |  |  |  |  |
|  | C1=0.1μg/mL | C2=1.0μg/mL | C3=5.0μg/mL | C4=10.0μg/mL |
| 1-Naphthaleneacetic acid |  |  |  |  |
|  | C1=0.1μg/mL | C2=1.0μg/mL | C3=5.0μg/mL | C4=10.0μg/mL |
| Indole-3-acetic acid |  |  |  |  |
|  | C1=0.1μg/mL | C2=1.0μg/mL | C3=5.0μg/mL | C4=10.0μg/mL |
| Salicylic acid |  |  |  |  |
|  | C1=0.1μg/mL | C2=1.0μg/mL | C3=5.0μg/mL | C4=10.0μg/mL |
| Coconut water |  |  |  |  |
|  | C1=1% | C2=3% | C3=7% | C4=10% |
| *Aloe vera* |  |  |  |  |
|  | C1=1% | C2=3% | C3=7% | C4=10% |
| Lentil sprout extract |  |  |  |  |
|  | C1=0.1μg/mL | C2=1.0μg/mL | C3=5.0μg/mL | C4=10.0μg/mL |
| Sargassum extract |  |  |  |  |
|  | C1=0.1μg/mL | C2=1.0μg/mL | C3=5.0μg/mL | C4=10.0μg/mL |
| **Wavelength** | | | | |
| Lights |  |  |  |  |
|  | L1=Blue light | L2=Red light | L3=Green light | L4=Yellow light |
| **CO_2_** | | | | |
| CO_2_ |  |  |  |  |
|  | T1=30s | T2=60s | T3=90s | T4=120s |

**H.** Effect of 7 culture conditions on the growth of *T. dimorphus.*

|  |  |  |  |
| --- | --- | --- | --- |
| Control | C1 - Coconut water 3% | C2 - Blue light (400-490nm) | C3 - 60s of CO_2_ |
|  |  |  |  |
| C4 - Coconut water 3% + Blue light (400-490nm) + 60s of CO_2_ | C5 - Coconut water 3% + Blue light (400-490nm) | C6 - Coconut water 3% + 60s of CO_2_ | C7 - Blue light (400-490nm) + 60s of CO_2_ |
