# Supplementary material for: Enhancing microalgal productivity through bioactive substances, light, and CO2
Source: PLoS One. 2026 Apr 27;21(4):e0338585. doi: 10.1371/journal.pone.0338585 (PMC13119868; doi:10.1371/journal.pone.0338585)

**A.** Comparison of the growth curve of *A. platensis* from the control vs. *Aloe vera* at 3%, for 30 days.


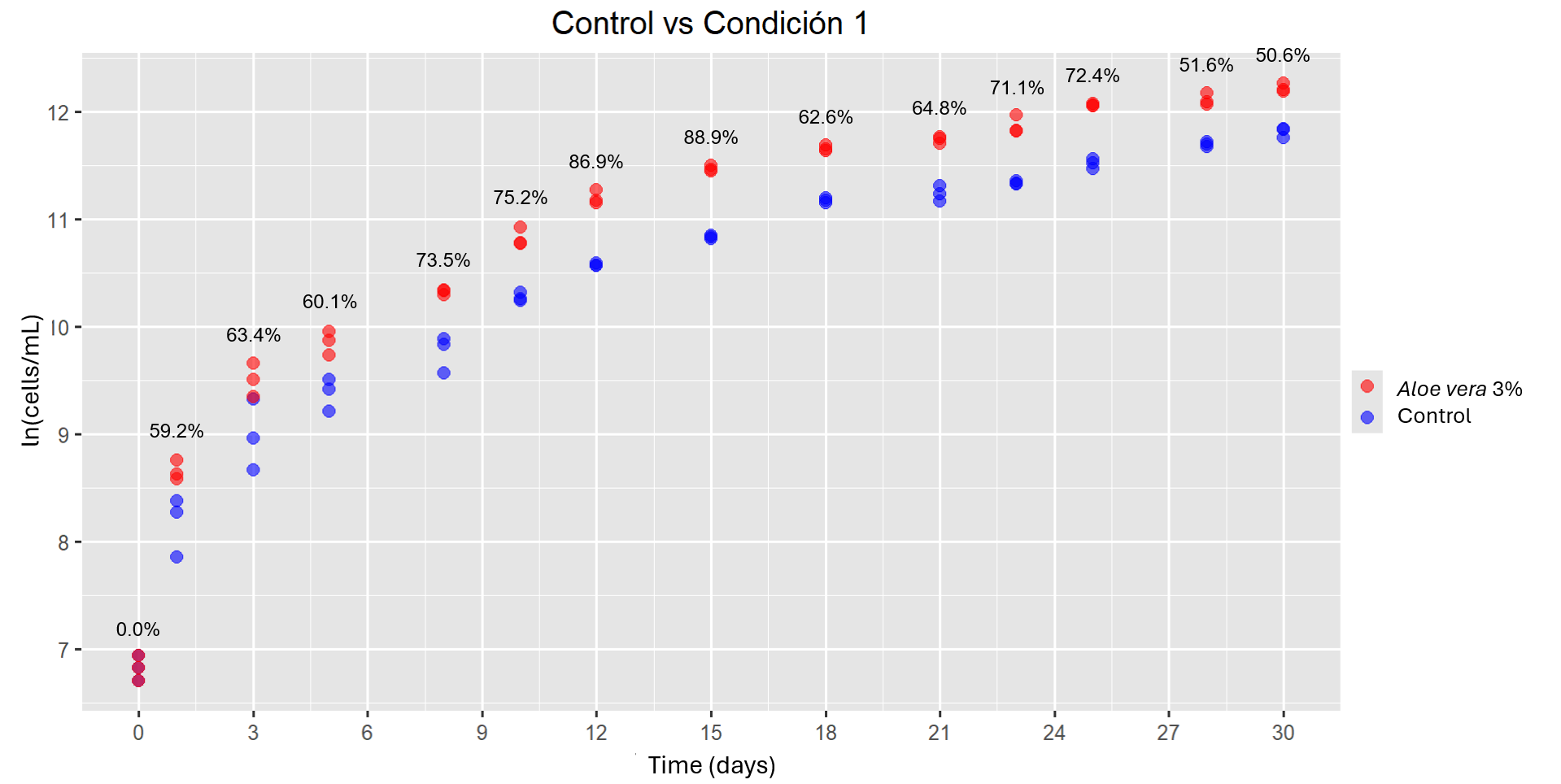


**B.** Comparison of the growth curve of *C. vulgaris* from the control vs. *Aloe vera* at 3%, for 30 days.

**
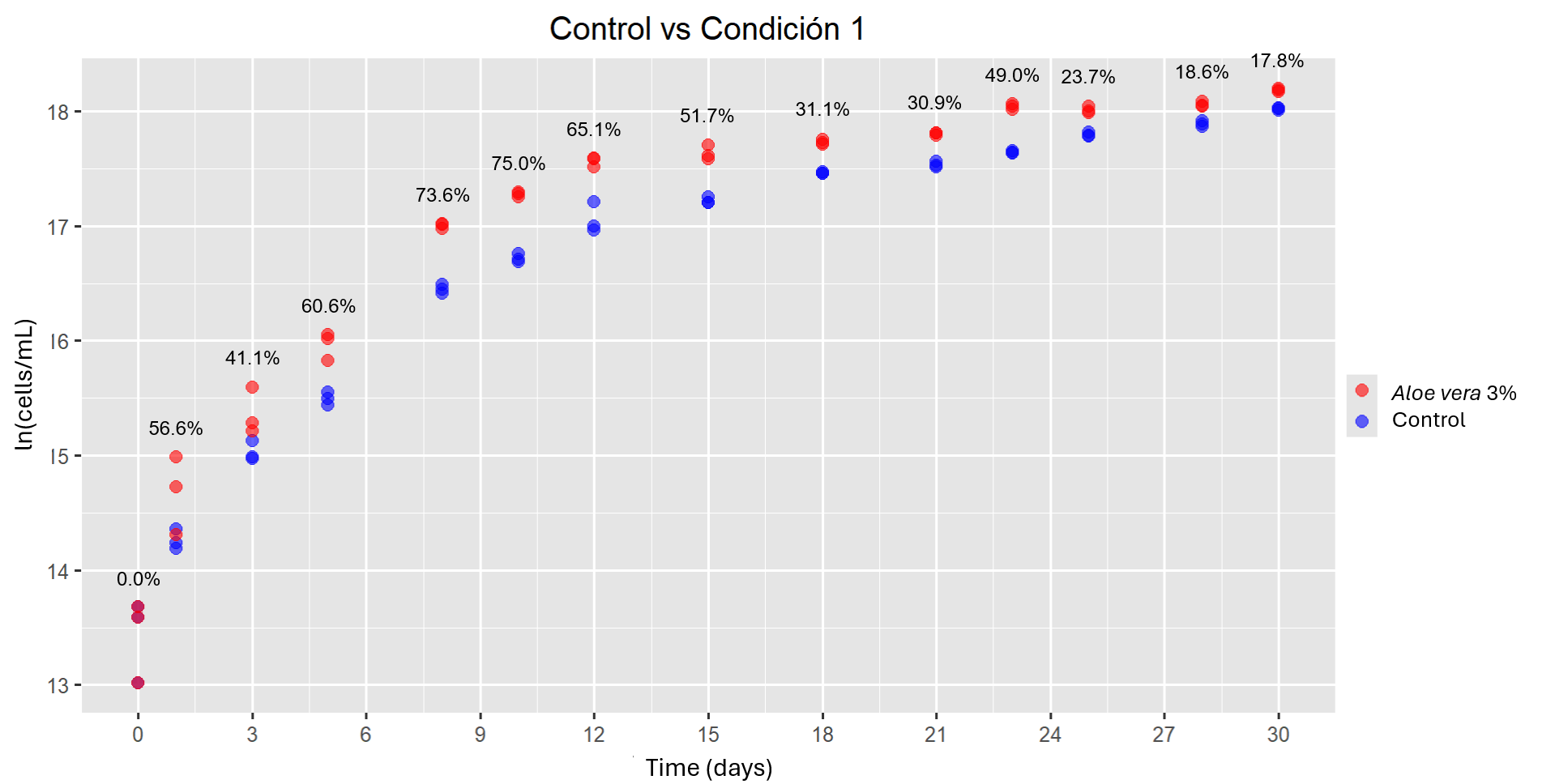
**

**C.** Comparison of the growth curve of *A. falcatus* from the control vs. *Aloe vera* at 1%, for 30 days.


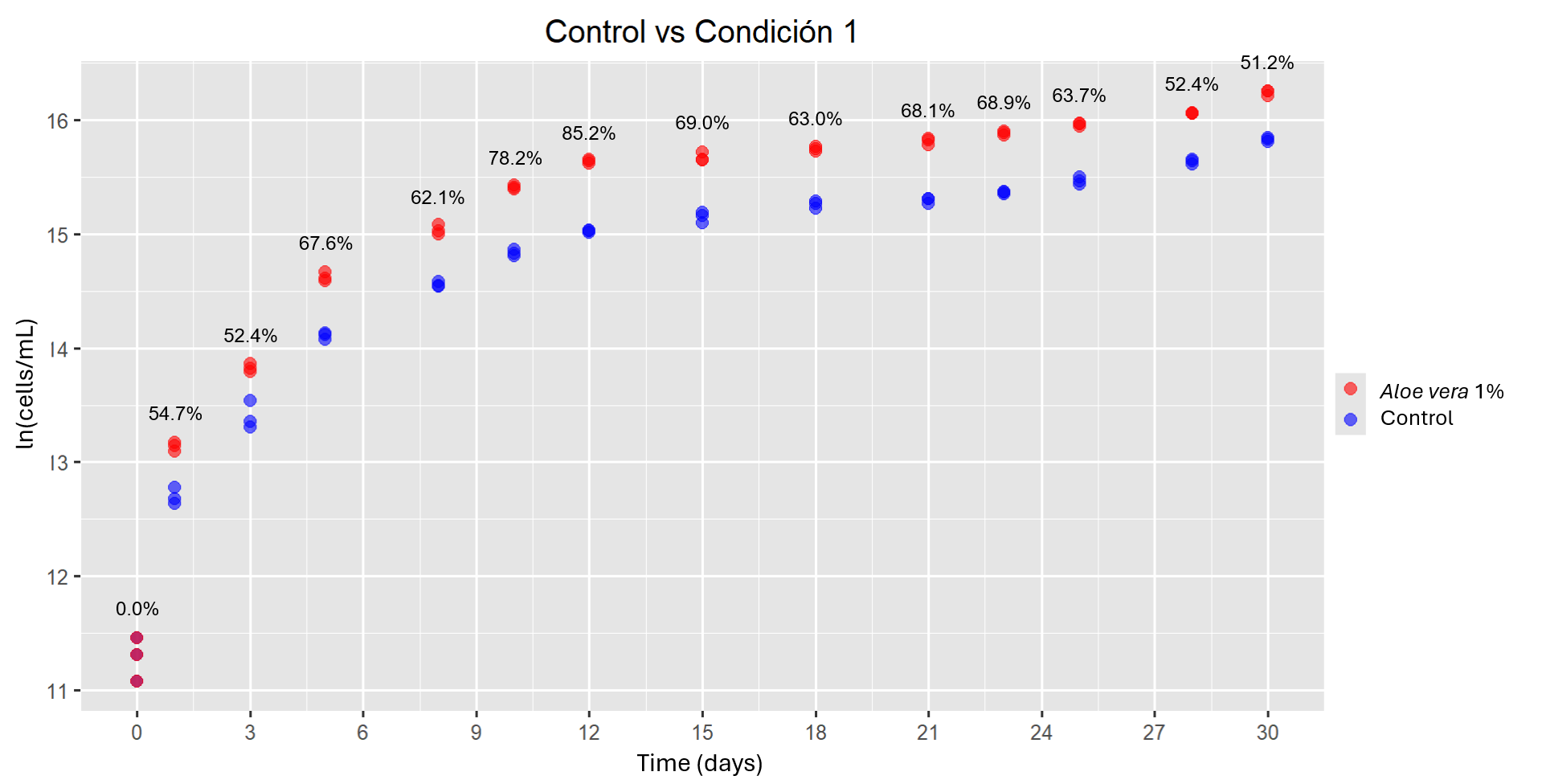


**D.** Comparison of the growth curve of *T. dimorphus* of control vs. 3% coconut water for 30 days.


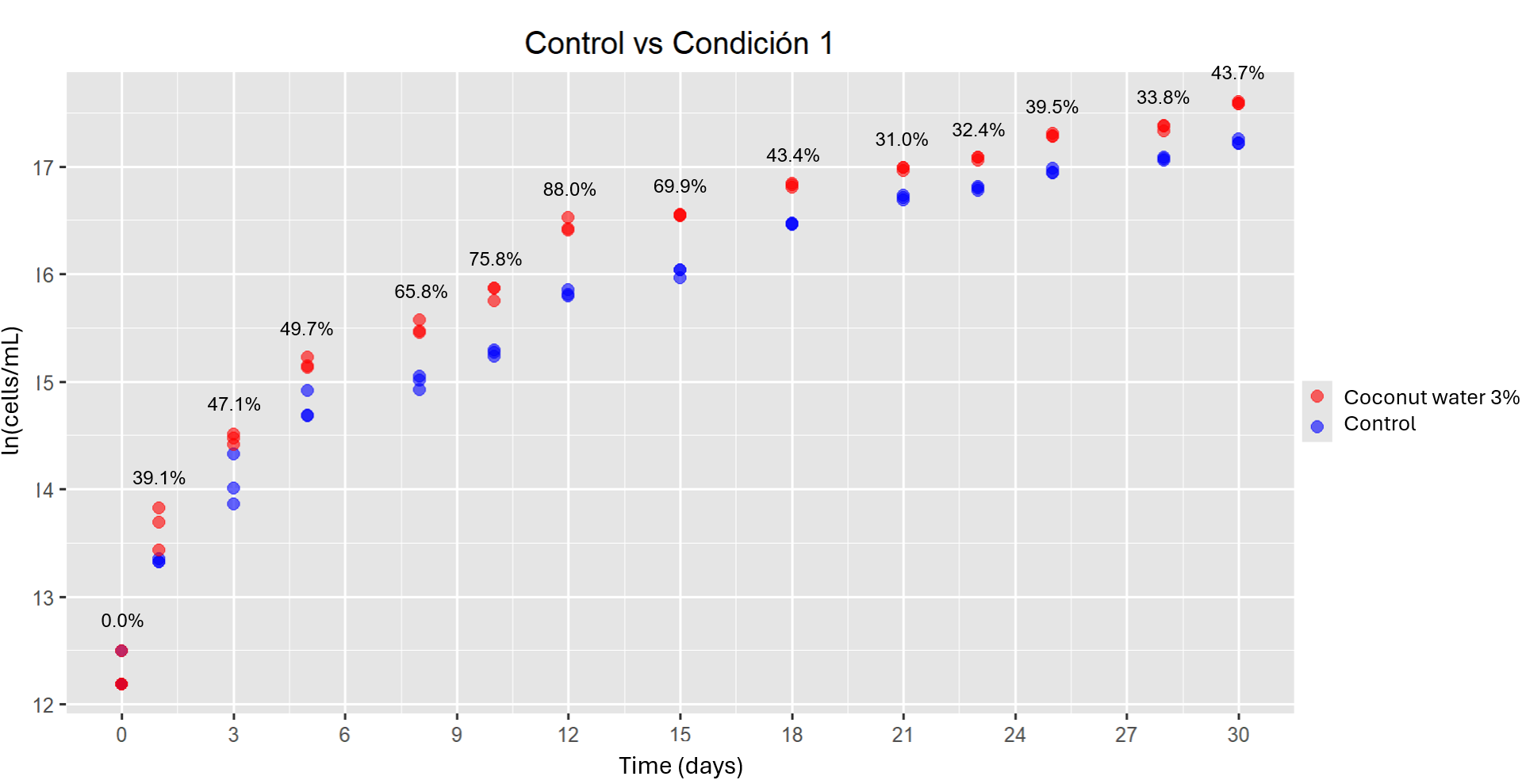

Supplement: S2 File — (DOCX) [file pone.0338585.s002.docx]
