# Supplementary material for: Enhancing microalgal productivity through bioactive substances, light, and CO2
Source: PLoS One. 2026 Apr 27;21(4):e0338585. doi: 10.1371/journal.pone.0338585 (PMC13119868; doi:10.1371/journal.pone.0338585)
Supplement: S3 File — (DOCX) [file pone.0338585.s003.docx]

**A.** Evaluation of statistical assumptions of linear models for the effect of 10 bioactive substances, wavelengths, CO_2_, and combined conditions on the growth of *A. platensis, C. vulgaris, A. falcatus* and *T. dimorphus.*

| **Microalgae** | **Condition Evaluated** | **Linear model (R^2^)** | **Assumptions** | | |
| --- | --- | --- | --- | --- | --- |
|  |  |  | **Normality** | **Independence** | **Homoscedasticity** |
| *A. platensis* | Bioactive substances | 0.9241 | 0.08055 | 0.8336 | 0,0007382 |
|  | Wavelengths | 0.9061 | 0.9198 | 0.5705 | 0.1218 |
|  | CO_2_ | 0.9361 | 0.6654 | 0.9385 | 0.1493 |
|  | Combined Conditions | 0.9845 | 0.835 | 0.6188 | 0.04758 |
| *C. vulgaris* | Bioactive substances | 0.9016 | 0.107 | 0.6822 | 0,0002689 |
|  | Wavelengths | 0.9896 | 0.3149 | 0.9821 | 0.6311 |
|  | CO_2_ | 0.9589 | 0.9430 | 0.9255 | 0.1550 |
|  | Combined Conditions | 0.9867 | 0.719 | 0.2634 | 0.09186 |
| *A. falcatus* | Bioactive substances | 0.9374 | 0.1895 | 0.4032 | 0.0001629 |
|  | Wavelengths | 0.9106 | 0.8144 | 0.09111 | 0.1108 |
|  | CO_2_ | 0.9665 | 0.8372 | 0.5004 | 0.07676 |
|  | Combined Conditions | 0.9984 | 0.996 | 0.5833 | 0.1272 |
| *T. dimorphus* | Bioactive substances | 0.8815 | 0.8334 | 0.9887 | 0.006517 |
|  | Wavelengths | 0.9714 | 0.9016 | 0.5287 | 0.163 |
|  | CO_2_ | 0.9251 | 0.6146 | 0.5935 | 0.09392 |
|  | Combined Conditions | 0.9928 | 0.3401 | 0.1599 | 0.09909 |

**B.** Effect of 10 bioactive substances with their 4 concentrations on the growth of *A. platensis, C. vulgaris, A. falcatus* and *T. dimorphus*.

| **Code** | **Substance** | **Concentration** | **Code** | **Trt** | **p-value** | **Effect** | **LSD** | **PIC (%)** |
| --- | --- | --- | --- | --- | --- | --- | --- | --- |
| ***A. platensis*** | | | | | | | | |
| SA | N-Butyryl-DL-homoserine lactone | 1.0μg/mL | C2 | 2 | 3.87e-12 | (+)*** | a | 94.4 |
| SH | *Aloe vera* | 3% | C2 | 30 | 6.38e-12 | (+)*** | a | 91.9 |
| SH | *Aloe vera* | 7% | C3 | 31 | 3.23e-11 | (+)*** | a | 88.1 |
| SC | Indole-3-butyric acid | 10.0μg/mL | C4 | 12 | 7.60e-10 | (+)*** | ab | 78.7 |
| SA | N-Butyryl-DL-homoserine lactone | 0.1μg/mL | C1 | 1 | 1.52e-07 | (+)*** | bc | 60.1 |
| SC | Indole-3-butyric acid | 5.0μg/mL | C3 | 11 | 6.94e-07 | (+)*** | bcd | 55.9 |
| SA | N-Butyryl-DL-homoserine lactone | 5.0μg/mL | C3 | 3 | 2.71e-06 | (+)*** | cde | 51.3 |
| SG | Coconut water | 3% | C2 | 26 | 6.46e-60 | (+)*** | cde | 47.7 |
| SG | Coconut water | 1% | C1 | 25 | 6.43e-06 | (+)*** | cde | 47.7 |
| SH | *Aloe vera* | 10% | C4 | 32 | 6.52e-06 | (+)*** | cdef | 47.2 |
| SC | Indole-3-butyric acid | 1.0μg/mL | C2 | 10 | 0.000507 | (+)*** | defg | 33.9 |
| SA | N-Butyryl-DL-homoserine lactone | 10.0μg/mL | C4 | 4 | 0.000855 | (+)*** | efg | 32.2 |
| SC | Indole-3-butyric acid | 0.1μg/mL | C1 | 9 | 0.001505 | (+)** | efgh | 32.0 |
| SJ | Sargassum extract | 5.0μg/mL | C3 | 39 | 0.005454 | (+)** | fghi | 27.3 |
| SG | Coconut water | 7% | C3 | 27 | 0.026756 | (+)* | ghij | 19.2 |
| SE | Indole-3-acetic acid | 10.0μg/mL | C4 | 20 | 0.120093 | (+) | hij | 13.6 |
| SD | 1-Naphthaleneacetic acid | 10.0μg/mL | C4 | 16 | 0.124063 | (+) | ij | 13.7 |
| SE | Indole-3-acetic acid | 0.1μg/mL | C1 | 17 | 0.209170 | (+) | ijk | 11.7 |
| SE | Indole-3-acetic acid | 5.0μg/mL | C3 | 19 | 0.340646 | (+) | jkl | 7.4 |
| SD | 1-Naphthaleneacetic acid | 1.0μg/mL | C2 | 14 | 0.358398 | (+) | jkl | 7.5 |
| SF | Salicylic acid | 10.0μg/mL | C4 | 24 | 0.422837 | (+) | jkl | 7.1 |
| SF | Salicylic acid | 1.0μg/mL | C2 | 22 | 0.462568 | (+) | jkl | 5.4 |
| Control | Control | Control | Control | 0 | Control | Control | klm | 0 |
| SE | Indole-3-acetic acid | 1.0μg/mL | C2 | 18 | 0.615982 | (-) | lmn | -4.2 |
| SD | 1-Naphthaleneacetic acid | 5.0μg/mL | C3 | 15 | 0.442967 | (-) | lmno | -6.9 |
| SF | Salicylic acid | 5.0μg/mL | C3 | 23 | 0.441753 | (-) | lmno | -7.3 |
| SB | L-Homoserine lactone hydrochloride | 10.0μg/mL | C4 | 8 | 0.228703 | (-) | mno | -9.2 |
| SJ | Sargassum extract | 1.0μg/mL | C2 | 38 | 0.098363 | (-). | nop | -13.4 |
| SF | Salicylic acid | 0.1μg/mL | C1 | 21 | 0.077761 | (-). | nop | -14.1 |
| SD | 1-Naphthaleneacetic acid | 0.1μg/mL | C1 | 13 | 0.071155 | (-). | nop | -14.5 |
| SH | *Aloe vera* | 1% | C1 | 29 | 0.055203 | (-). | nopq | -15.6 |
| SI | Lentil sprout extract | 10.0μg/mL | C4 | 36 | 0.046285 | (-)* | nopq | -15.6 |
| SB | L-Homoserine lactone hydrochloride | 0.1μg/mL | C1 | 5 | 0.023599 | (-)* | nopq | -18.5 |
| SJ | Sargassum extract | 10.0μg/mL | C4 | 40 | 0.012814 | (-)* | opq | -19.0 |
| SB | L-Homoserine lactone hydrochloride | 1.0μg/mL | C2 | 6 | 0.003019 | (-)** | pq | -23.2 |
| SI | Lentil sprout extract | 5.0μg/mL | C3 | 35 | 0.002065 | (-)** | pq | -23.6 |
| SG | Coconut water | 10% | C4 | 28 | 0.000711 | (-)*** | qr | -26.8 |
| SI | Lentil sprout extract | 1.0μg/mL | C2 | 34 | 1.80e-06 | (-)*** | rs | -35.5 |
| SI | Lentil sprout extract | 0.1μg/mL | C1 | 33 | 6.07e-10 | (-)*** | st | -44.2 |
| SJ | Sargassum extract | 0.1μg/mL | C1 | 37 | 4.09e-10 | (-)*** | st | -44-3 |
| SB | L-Homoserine lactone hydrochloride | 5.0μg/mL | C3 | 7 | 7.65e-12 | (-)*** | t | -48.8 |
| ***C. vulgaris*** | | | | | | | | |
| SH | *Aloe vera* | 3% | C2 | 30 | <2e-16 | (+)*** | a | 76.2 |
| SB | L-Homoserine lactone hydrochloride | 10.0μg/mL | C4 | 8 | <2e-16 | (+)*** | a | 76.0 |
| SG | Coconut water | 1% | C1 | 25 | <2e-16 | (+)*** | a | 73.6 |
| SI | Lentil sprout extract | 1.0μg/mL | C2 | 34 | <2e-16 | (+)*** | a | 69.9 |
| SB | L-Homoserine lactone hydrochloride | 5.0μg/mL | C3 | 7 | 4.40e-16 | (+)*** | ab | 61.7 |
| SA | N-Butyryl-DL-homoserine lactone | 10.0μg/mL | C4 | 4 | 1.62e-14 | (+)*** | abc | 56.8 |
| SH | *Aloe vera* | 1% | C1 | 29 | 1.71e-12 | (+)*** | bcd | 48.7 |
| SB | L-Homoserine lactone hydrochloride | 1.0μg/mL | C2 | 6 | 2.49e-12 | (+)*** | bcd | 48.6 |
| SJ | Sargassum extract | 1.0μg/mL | C2 | 38 | 3.60e-12 | (+)*** | bcd | 47.5 |
| SH | *Aloe vera* | 7% | C3 | 31 | 5.69e-10 | (+)*** | cde | 40.0 |
| SG | Coconut water | 3% | C2 | 26 | 1.37e-09 | (+)*** | cdef | 38.7 |
| SI | Lentil sprout extract | 10.0μg/mL | C4 | 36 | 1.64e-09 | (+)*** | cdef | 38.5 |
| SJ | Sargassum extract | 5.0μg/mL | C3 | 39 | 7.03e-09 | (+)*** | defg | 36.5 |
| SI | Lentil sprout extract | 5.0μg/mL | C3 | 35 | 2.00e-08 | (+)*** | defgh | 34.8 |
| SA | N-Butyryl-DL-homoserine lactone | 5.0μg/mL | C3 | 3 | 2.58e-08 | (+)*** | defghi | 34.4 |
| SE | Indole-3-acetic acid | 5.0μg/mL | C3 | 19 | 7.33e-06 | (+)*** | efghij | 25.9 |
| SJ | Sargassum extract | 0.1μg/mL | C1 | 37 | 1.71e-05 | (+)*** | efghij | 25.8 |
| SC | Indole-3-butyric acid | 0.1μg/mL | C1 | 9 | 2.01e-05 | (+)*** | efghij | 24.4 |
| SA | N-Butyryl-DL-homoserine lactone | 1.0μg/mL | C2 | 2 | 4.14e-05 | (+)*** | fghij | 23.2 |
| SE | Indole-3-acetic acid | 1.0μg/mL | C2 | 18 | 0.000180 | (+)*** | ghijk | 21.7 |
| SF | Salicylic acid | 1.0μg/mL | C2 | 22 | 0.000419 | (+)*** | hijk | 20.6 |
| SA | N-Butyryl-DL-homoserine lactone | 0.1μg/mL | C1 | 1 | 0.00463 | (+)*** | ijk | 19.4 |
| SB | L-Homoserine lactone hydrochloride | 0.1μg/mL | C1 | 5 | 0.000777 | (+)*** | jk | 18.4 |
| SF | Salicylic acid | 0.1μg/mL | C1 | 21 | 0.001430 | (+)** | jk | 17.3 |
| SC | Indole-3-butyric acid | 10.0μg/mL | C4 | 12 | 0.001613 | (+)** | jk | 17.2 |
| SD | 1-Naphthaleneacetic acid | 1.0μg/mL | C2 | 14 | 0.007764 | (+)** | jkl | 14.1 |
| SC | Indole-3-butyric acid | 5.0μg/mL | C3 | 11 | 0.016591 | (+)* | jklm | 12.6 |
| SE | Indole-3-acetic acid | 0.1μg/mL | C1 | 17 | 0.150958 | (+) | klmn | 8.3 |
| SG | Coconut water | 7% | C3 | 27 | 0.471084 | (+) | lmn | 3.5 |
| SF | Salicylic acid | 5.0μg/mL | C3 | 23 | 0.595894 | (+) | lmn | 2.6 |
| SH | *Aloe vera* | 10% | C4 | 32 | 0.875157 | (+) | mn | 0.7 |
| Control | Control | Control | Control | 0 | Control | Control | n | 0 |
| SJ | Sargassum extract | 10.0μg/mL | C4 | 40 | 0.781286 | (-) | n | -1.4 |
| SD | 1-Naphthaleneacetic acid | 5.0μg/mL | C3 | 15 | 0.535558 | (-) | n | -2.8 |
| SD | 1-Naphthaleneacetic acid | 10.0μg/mL | C4 | 16 | 0.455786 | (-) | n | -3.6 |
| SC | Indole-3-butyric acid | 1.0μg/mL | C2 | 10 | 0.389589 | (-) | n | -5.0 |
| SD | 1-Naphthaleneacetic acid | 0.1μg/mL | C1 | 13 | 0.299182 | (-) | no | -4.0 |
| SI | Lentil sprout extract | 0.1μg/mL | C1 | 33 | 0.000603 | (-)*** | op | -15.8 |
| SE | Indole-3-acetic acid | 10.0μg/mL | C4 | 20 | 7.26e-05 | (-)*** | p | -18.3 |
| SF | Salicylic acid | 10.0μg/mL | C4 | 24 | 3.35e-11 | (-)*** | q | -30.7 |
| SG | Coconut water | 10% | C4 | 28 | 1e-13 | (-)*** | q | -34.7 |
| ***A. falcatus*** | | | | | | | | |
| SH | *Aloe vera* | 1% | C1 | 29 | 2e-16 | (+)*** | a | 97.4 |
| SG | Coconut water | 1% | C1 | 25 | 2.22e-13 | (+)*** | ab | 76.6 |
| SH | *Aloe vera* | 3% | C2 | 30 | 5.93e-13 | (+)*** | ab | 74.3 |
| SG | Coconut water | 3% | C2 | 26 | 2.86e-11 | (+)*** | bc | 65.0 |
| SH | *Aloe vera* | 7% | C3 | 31 | 2.23e-10 | (+)*** | bcd | 60.4 |
| SJ | Sargassum extract | 5.0μg/mL | C3 | 39 | 7.51e-08 | (+)*** | cde | 47.3 |
| SH | *Aloe vera* | 10% | C4 | 32 | 6.92e-06 | (+)** | def | 37.3 |
| SJ | Sargassum extract | 1.0μg/mL | C2 | 38 | 7.51e-08 | (+)** | efg | 31.9 |
| SB | L-Homoserine lactone hydrochloride | 10.0μg/mL | C4 | 8 | 0.000132 | (+)*** | efg | 30.6 |
| SF | Salicylic acid | 1.0μg/mL | C2 | 22 | 0.000984 | (+)*** | efgh | 25.4 |
| SE | Indole-3-acetic acid | 1.0μg/mL | C2 | 18 | 0.001096 | (+)** | efgh | 25.0 |
| SE | Indole-3-acetic acid | 5.0μg/mL | C3 | 19 | 0.002612 | (+)** | fghi | 22.7 |
| SE | Indole-3-acetic acid | 0.1μg/mL | C1 | 17 | 0.030350 | (+)** | fghi | 22.5 |
| SD | 1-Naphthaleneacetic acid | 1.0μg/mL | C2 | 14 | 0.016903 | (+)* | fghi | 17.6 |
| SF | Salicylic acid | 10.0μg/mL | C4 | 24 | 0.055892 | (+). | ghij | 13.6 |
| SF | Salicylic acid | 5.0μg/mL | C3 | 23 | 0.164453 | (+) | hijk | 9.6 |
| SD | 1-Naphthaleneacetic acid | 0.1μg/mL | C1 | 13 | 0.212074 | (+) | hijk | 8.9 |
| SA | N-Butyryl-DL-homoserine lactone | 1.0μg/mL | C2 | 2 | 0.256262 | (+) | hijjkl | 7.8 |
| SB | L-Homoserine lactone hydrochloride | 1.0μg/mL | C2 | 6 | 0.418621 | (+) | ijklm | 5.4 |
| SC | Indole-3-butyric acid | 0.1μg/mL | C1 | 9 | 0.943732 | (+) | jklm | 1.8 |
| Control | Control | Control | Control | 0 | Control | Control | jklm | 0.0 |
| SC | Indole-3-butyric acid | 1.0μg/mL | C2 | 10 | 0.991279 | (-) | jklmn | -0.1 |
| SF | Salicylic acid | 0.1μg/mL | C1 | 21 | 0.932604 | (-) | jklmn | -0.5 |
| SB | L-Homoserine lactone hydrochloride | 0.1μg/mL | C1 | 5 | 0.742106 | (-) | jklmno | -2.1 |
| SE | Indole-3-acetic acid | 10.0μg/mL | C4 | 20 | 0.649355 | (-) | jklmno | -3.0 |
| SJ | Sargassum extract | 0.1μg/mL | C1 | 37 | 0.469808 | (-) | klmno | -4.5 |
| SA | N-Butyryl-DL-homoserine lactone | 5.0μg/mL | C3 | 3 | 0.956110 | (-) | klmno | -5.1 |
| SJ | Sargassum extract | 10.0μg/mL | C4 | 40 | 0.416331 | (-) | klmno | -5.4 |
| SI | Lentil sprout extract | 0.1μg/mL | C1 | 33 | 0.308190 | (-) | klmno | -6.1 |
| SA | N-Butyryl-DL-homoserine lactone | 0.1μg/mL | C1 | 1 | 0.192215 | (-) | lmno | -8.4 |
| SB | L-Homoserine lactone hydrochloride | 5.0μg/mL | C3 | 7 | 0.186034 | (-) | lmno | -8.5 |
| SA | N-Butyryl-DL-homoserine lactone | 10.0μg/mL | C4 | 4 | 0.132924 | (-) | mnop | -9.7 |
| SI | Lentil sprout extract | 1.0μg/mL | C2 | 34 | 0.039608 | (-)* | nop | -12.5 |
| SD | 1-Naphthaleneacetic acid | 5.0μg/mL | C3 | 15 | 0.010906 | (-)* | opq | -15.9 |
| SI | Lentil sprout extract | 5.0μg/mL | C3 | 35 | 0.000158 | (-)*** | pq | -20.9 |
| SG | Coconut water | 7% | C3 | 27 | 0.000181 | (-)*** | pq | -22.8 |
| SD | 1-Naphthaleneacetic acid | 10.0μg/mL | C4 | 16 | 0.015175 | (-)*** | q | -28.5 |
| SC | Indole-3-butyric acid | 5.0μg/mL | C3 | 11 | 2e-16 | (-)*** | r | -52.4 |
| SC | Indole-3-butyric acid | 10.0μg/mL | C4 | 12 | 2e-16 | (-)*** | s | -64.5 |
| SI | Lentil sprout extract | 10.0μg/mL | C4 | 36 | 2e-16 | (-)*** | st | -67.2 |
| SG | Coconut water | 10% | C4 | 28 | <2e-16 | (-)*** | t | -71.3 |
| ***T. dimorphus*** | | | | | | | | |
| SD | 1-Naphthaleneacetic acid | 10.0μg/mL | C4 | 16 | 2.39e-16 | (+)*** | a | 138.1 |
| SC | Indole-3-butyric acid | 10.0μg/mL | C4 | 12 | 1.91e-13 | (+)*** | ab | 115.5 |
| SD | 1-Naphthaleneacetic acid | 5.0μg/mL | C3 | 15 | 4.64e-13 | (+)*** | ab | 111.0 |
| SG | Coconut water | 3% | C2 | 26 | 1.90e-11 | (+)*** | bc | 93.8 |
| SF | Salicylic acid | 10.0μg/mL | C4 | 24 | 4.27e-11 | (+)*** | bc | 91.6 |
| SC | Indole-3-butyric acid | 5.0μg/mL | C3 | 11 | 4.44e-11 | (+)*** | bc | 91.4 |
| SA | N-Butyryl-DL-homoserine lactone | 0.1μg/mL | C1 | 1 | 1.30e-18 | (+)*** | cd | 71.6 |
| SA | N-Butyryl-DL-homoserine lactone | 1.0μg/mL | C2 | 2 | 7.71e-08 | (+)*** | cde | 66.0 |
| SA | N-Butyryl-DL-homoserine lactone | 5.0μg/mL | C3 | 3 | 2.31e-07 | (+)*** | def | 62.2 |
| SJ | Sargassum extract | 10.0μg/mL | C4 | 40 | 3.92e-07 | (+)*** | def | 60.7 |
| SE | Indole-3-acetic acid | 5.0μg/mL | C3 | 19 | 6.63e-07 | (+)*** | def | 58.9 |
| SE | Indole-3-acetic acid | 10.0μg/mL | C4 | 20 | 9.76e-07 | (+)*** | def | 58.0 |
| SA | N-Butyryl-DL-homoserine lactone | 10.0μg/mL | C4 | 4 | 1.21e-06 | (+)*** | def | 58.0 |
| SJ | Sargassum extract | 5.0μg/mL | C3 | 39 | 2.81e-06 | (+)*** | defg | 55.8 |
| SF | Salicylic acid | 5.0μg/mL | C3 | 23 | 7.45e-06 | (+)*** | defgh | 50.9 |
| SI | Lentil sprout extract | 0.1μg/mL | C1 | 33 | 7.79e-06 | (+)*** | defgh | 50.9 |
| SJ | Sargassum extract | 1.0μg/mL | C2 | 38 | 1.63e-05 | (+)*** | defgh | 49.6 |
| SD | 1-Naphthaleneacetic acid | 1.0μg/mL | C2 | 14 | 6.68e-05 | (+)*** | efgh | 43.3 |
| SI | Lentil sprout extract | 1.0μg/mL | C2 | 34 | 0.000163 | (+)*** | efgh | 42.9 |
| SH | *Aloe vera* | 7% | C3 | 31 | 0.000199 | (+)*** | fgh | 39.8 |
| SI | Lentil sprout extract | 5.0μg/mL | C3 | 35 | 0.000261 | (+)*** | fghi | 39.2 |
| SG | Coconut water | 7% | C3 | 27 | 0.000284 | (+)*** | fghi | 38.3 |
| SD | 1-Naphthaleneacetic acid | 0.1μg/mL | C1 | 13 | 0.001772 | (+)** | ghij | 33.4 |
| SG | Coconut water | 1% | C1 | 25 | 0.002504 | (+)** | ghij | 30.0 |
| SJ | Sargassum extract | 0.1μg/mL | C1 | 37 | 0.006898 | (+)** | hij | 28.2 |
| SE | Indole-3-acetic acid | 0.1μg/mL | C1 | 17 | 0.049093 | (+)* | ijk | 19.2 |
| SE | Indole-3-acetic acid | 1.0μg/mL | C2 | 18 | 0.061186 | (+). | jk | 19.1 |
| SI | Lentil sprout extract | 10.0μg/mL | C4 | 36 | 0.093697 | (+). | jk | 17.7 |
| SH | *Aloe vera* | 3% | C2 | 30 | 0.102965 | (+) | jk | 17.1 |
| SC | Indole-3-butyric acid | 0.1μg/mL | C1 | 9 | 0.131075 | (+) | jk | 16.8 |
| SB | L-Homoserine lactone hydrochloride | 0.1μg/mL | C1 | 5 | 0.257740 | (+) | kl | 11.4 |
| SF | Salicylic acid | 1.0μg/mL | C2 | 22 | 0.296914 | (+) | klm | 9.3 |
| SC | Indole-3-butyric acid | 1.0μg/mL | C2 | 10 | 0.487492 | (+) | klmn | 6.6 |
| SB | L-Homoserine lactone hydrochloride | 5.0μg/mL | C3 | 7 | 0.565437 | (+) | klmn | 5.1 |
| SH | *Aloe vera* | 1% | C1 | 29 | 0.618877 | (+) | klmn | 4.7 |
| SB | L-Homoserine lactone hydrochloride | 1.0μg/mL | C2 | 6 | 0.839650 | (+) | klmn | 1.5 |
| Control | Control | Control | Control | 0 | Control | Control | lmn | 0 |
| SF | Salicylic acid | 0.1μg/mL | C1 | 21 | 0.373760 | (-) | mn | -6.4 |
| SG | Coconut water | 10% | C4 | 28 | 0.292994 | (-) | n | -8.9 |
| SH | *Aloe vera* | 10% | C4 | 32 | 0.253702 | (-) | n | -9.3 |
| SB | L-Homoserine lactone hydrochloride | 10.0μg/mL | C4 | 8 | 1.09e-06 | (-)*** | o | -36.4 |

Trt: treatment; LSD: Least Significant Difference test; PIC: percentage of growth induction; (+): positive effect; (-): negative effect. The following were considered statistically significant: p≤0.0001(***); p≤0.001(**); p≤0.01(*); p≤0.05 (.); and p≤0.1() as non-significant.

**C.** Effect of 4 wavelengths on the growth of *A. platensis, C. vulgaris, A. falcatus* and *T. dimorphus*.

| **Microalgae** | **Code** | **Wavelength** | **p-value** | **Effect** | **LSD** | **PIC (%)** |
| --- | --- | --- | --- | --- | --- | --- |
| *A. platensis* | L2 | Red light | 0.000125 | (+)*** | a | 49.2 |
|  | L4 | Yellow light | 0.031155 | (+)* | b | 18.1 |
|  | Control | Control | Control | Control | c | 0 |
|  | L3 | Green light | 0.512242 | (-) | c | -3.8 |
|  | L1 | Blue light | 0.000367 | (-)*** | d | -29.2 |
| *C. vulgaris* | L1 | Blue light | 2.47e-08 | (+)*** | a | 57.7 |
|  | Control | Control | Control | Control | b | 0 |
|  | L4 | Yellow light | 0.00661 | (-)** | c | -9.5 |
|  | L3 | Green light | 6.19e-08 | (-)*** | d | -33.8 |
|  | L2 | Red light | 1.16e-08 | (-)*** | e | -38.9 |
| *A. falcatus* | L2 | Red light | 0.00177 | (+)** | a | 20.8 |
|  | Control | Control | Control | Control | ab | 0 |
|  | L1 | Blue light | 0.33418 | (-) | b | -4.4 |
|  | L3 | Green light | 0.36103 | (-) | b | -4.3 |
|  | L4 | Yellow light | 1.62e-05 | (-)*** | c | -29.1 |
| *T. dimorphus* | L1 | Blue light | 1.01e-06 | (+)*** | a | 31.5 |
|  | Control | Control | Control | Control | b | 0 |
|  | L4 | Yellow light | 0.0209 | (-)* | c | -6.9 |
|  | L3 | Green light | 3.40e-05 | (-)*** | d | -16.8 |
|  | L2 | Red light | 3.12e-06 | (-)*** | d | -21.5 |

LSD: Least Significant Difference test; PIC: percentage of growth induction; (+): positive effect; (-): negative effect. The following were considered statistically significant: p≤0.0001(***); p≤0.001(**); p≤0.01(*); p≤0.05 (.); and p≤0.1() were considered non-significant.

**D.** Effect of 4 CO₂ injection times on the growth of *A. platensis, C. vulgaris, A. falcatus* and *T. dimorphus*.

| **Microalgae** | **Code** | **CO_2_ injection times (s)** | **p-value** | **Effect** | **LSD** | **PIC (%)** | **Fi (gCO_2_)** |
| --- | --- | --- | --- | --- | --- | --- | --- |
| *A. platensis* | T2 | 60 | 1.12e-07 | (+)*** | a | 41.2 | 0.230 |
|  | T3 | 90 | 2.44e-05 | (+)*** | b | 21.1 | 0.204 |
|  | T1 | 30 | 2.39e-05 | (+)*** | b | 21.1 | 0.202 |
|  | T4 | 120 | 0.0295 | (+)* | c | 6.8 | 0.168 |
|  | Control | 0 | Control | Control | c | 0 | 0.162 |
| *C. vulgaris* | T2 | 60 | 1.67e-08 | (+)*** | a | 73.5 | 0.384 |
|  | T3 | 90 | 1.34e-05 | (+)*** | b | 30.6 | 0.296 |
|  | T1 | 30 | 1.60e-05 | (+)*** | b | 30.0 | 0.295 |
|  | T4 | 120 | 0.1820 | (+) | c | 4.9 | 0.239 |
|  | Control | 0 | Control | Control | c | 0 | 0.218 |
| *A. falcatus* | T1 | 30 | 6.38e-08 | (+)*** | a | 53.5 | 0.271 |
|  | T2 | 60 | 5.34e-07 | (+)*** | b | 40.7 | 0.232 |
|  | T3 | 90 | 7.55e-07 | (+)*** | b | 38.9 | 0.219 |
|  | Control | 0 | Control | Control | c | 0 | 0.170 |
|  | T4 | 120 | 0.166 | (-) | c | -4.4 | 0.154 |
| *T. dimorphus* | T2 | 60 | 0.000824 | (+)*** | a | 21.7 | 0.196 |
|  | Control | Control | Control | Control | b | 0 | 0.153 |
|  | T3 | 90 | 0.008011 | (-)** | c | -12.9 | 0.124 |
|  | T1 | 30 | 0.007142 | (-)** | c | -13.0 | 0.123 |
|  | T4 | 120 | 1.2e-05 | (-)*** | d | -28.4 | 0.096 |

LSD: Least Significant Difference test; PIC: percentage growth induction; (+): positive effect; (-): negative effect. The following were considered statistically significant: p≤0.0001(***); p≤0.001(**); p≤0.01(*); p≤0.05 (.); and p≤0.1() as non-significant. Fi: CO_2_ fixation for day i.

**E.** Effect of 7 culture conditions on the growth of *A. platensis, C. vulgaris, A. falcatus* and *T. dimorphus.*

| **Microalgae** | **Code** | **Condition** | **p-value** | **Effect** | **LSD** | **PIC (%)** |
| --- | --- | --- | --- | --- | --- | --- |
| *A. platensis* | C1 | *Aloe vera* 3% | <2.14e-10 | (+)*** | a | 85.3 |
|  | C2 | Red light (600-700nm) | 4.49e-08 | (+)*** | b | 52.9 |
|  | C3 | 60s of CO_2_ | 1.43e-06 | (+)*** | c | 38.7 |
|  | C6 | *Aloe vera* 3% + 60s of CO_2_ | 4.53e-05 | (+)*** | c | 27.6 |
|  | C5 | *Aloe vera* 3%+ Red light (600-700nm) | 0.0363 | (+)* | d | 10.6 |
|  | Control | Control | Control | Control | e | 0 |
|  | C7 | Red light (600-700nm) + 60s of CO_2_ | 1.73e-09 | (-)*** | f | -41.4 |
|  | C4 | *Aloe vera* 3% + Red light (600-700nm) + 60s of CO_2_ | 8.03e-11 | (-)*** | g | -48.0 |
| *C. vulgaris* | C1 | *Aloe vera* 3% | 2.46e-12 | (+)*** | a | 69.2 |
|  | C3 | 60s of CO_2_ | 1.51e-12 | (+)*** | a | 66.5 |
|  | C2 | Blue light (400-490nm) | 3.33e-11 | (+)*** | b | 53.6 |
|  | C5 | *Aloe vera* 3% + Blue light (400-490nm) | 0.0124 | (+)* | c | 8.0 |
|  | C6 | *Aloe vera* 3% + 60s of CO_2_ | 0.0660 | (+). | cd | 5.5 |
|  | Control | Control | Control | Control | de | 0 |
|  | C4 | *Aloe vera* 3% + Blue light (400-490nm) + 60s of CO_2_ | 0.7516 | (-) | e | -0.8 |
|  | C7 | Blue light (400-490nm) + 60s of CO_2_ | 3.16e-09 | (-)*** | f | -27.1 |
| *A. falcatus* | C1 | *Aloe vera* 1% | 3.95e-15 | (+)*** | a | 87.7 |
|  | C3 | 30 s de CO_2_ | 9.90e-12 | (+)*** | b | 46.2 |
|  | C6 | *Aloe vera* 1% + 30s of CO_2_ | 9.23e-09 | (+)*** | c | 27.1 |
|  | C2 | Red light (600-700nm) | 3.65e-06 | (+)*** | d | 16.5 |
|  | Control | Control | Control | Control | e | 0 |
|  | C4 | *Aloe vera* 1% + Red light (600-700nm) + 30s of CO_2_ | <2e-16 | (-)*** | f | -58.8 |
|  | C5 | *Aloe vera* 1% + Red light (600-700nm) | <2e-16 | (-)*** | f | -60.3 |
|  | C7 | Red light (600-700nm) + 30s of CO_2_ | <2e-16 | (-)*** | g | -71.9 |
| *T. dimorphus* | C1 | Coconut water 3% | 2.50e-10 | (+)*** | a | 80.5 |
|  | C2 | Blue light (400-490nm) | 3.59e-05 | (+)*** | b | 27.2 |
|  | C3 | 60s of CO_2_ | 0.00112 | (+)** | b | 18.3 |
|  | Control | Control | Control | Control | c | 0 |
|  | C6 | Coconut water 3% + 60s of CO_2_ | 1.64e-07 | (-)*** | d | -31.1 |
|  | C7 | Blue light (400-490nm) + 60s of CO_2_ | 2.63e-09 | (-)*** | e | -39.6 |
|  | C5 | Coconut water 3% + Blue light (400-490nm) | 3.66e-11 | (-)*** | f | -48.9 |
|  | C4 | Coconut water 3% + Blue light (400-490nm) + 60s of CO_2_ | <2e-16 | (-)*** | g | -76.7 |

LSD: Least Significant Difference test; PIC: percentage growth induction; (+): positive effect; (-): negative effect. Statistically significant measurements were considered: p≤0.0001 (***); p≤0.001 (**); p≤0.01 (*); p≤0.05 (.); and non-significant measurements were considered: p≤0.1().
