# Supplementary material for: Enhancing microalgal productivity through bioactive substances, light, and CO2
Source: PLoS One. 2026 Apr 27;21(4):e0338585. doi: 10.1371/journal.pone.0338585 (PMC13119868; doi:10.1371/journal.pone.0338585)
Supplement: Attachment — Submitted filename: Response to Reviewers.docx [file pone.0338585.s005.docx]

**Requeriment (March 5, 2026)**

This work was developed as part of an initial research project funded by Minciencias (Colombia) (COD 1115-914-91810, Ct 111-2022). However, the present study did not receive specific funding and is therefore not included in the acknowledgements.

**Editorial requirement:**

Figure 1 has been revised. The figure corresponds to an original photograph taken by the authors during their own experimental assays; therefore, no third-party copyrighted material is included.

**Reviewer #1:**

The manuscript presents a comprehensive experimental study systematically evaluating the effects of bioactive substances, light wavelength, and CO₂ injection regimes—both individually and in combination—on the growth of four microalgae/cyanobacterium of biotechnological interest (*Arthrospira platensis, Chlorella vulgaris, Ankistrodesmus falcatus,* and *Tetradesmus dimorphus*).

The inclusion of low-cost alternatives (such as Aloe vera and coconut water) is relevant from an applied perspective, and all data are fully available in the supplementary files, in accordance with the journal’s data availability requirements. Despite its experimental merit, the manuscript presents several important issues that need to be addressed before it can be considered for publication, including: Textual clarity and English quality: There are recurrent grammatical errors, inappropriate use of prepositions, agreement issues, and long or confusing sentence constructions. In several sections, the English does not fully compromise comprehension, but it does reduce scientific clarity, particularly in the Introduction and Discussion. Conceptual coherence between objectives, methods, and conclusions: The stated objective refers to growth “and/or nutritional quality,” but the nutritional analysis is limited and applied only to selected conditions. While the conclusions emphasize growth, the nutritional data show only modest changes. Statistical interpretation and risk of inflated significance: The extensive use of the LSD test in an experimental design involving many treatments increases the risk of Type I error. Although statistical assumptions were validated, the choice of the LSD test should be more clearly justified given the large number of comparisons. The specific points that require clarification and revision are detailed below.

**Abstract**

**1. The abstract presents a large number of percentage values, making it dense and difficult to read. Reducing the number of quantitative examples and retaining only the most representative ones is recommended.**

The abstract has been revised to substantially reduce the number of quantitative values, retaining only representative trends and emphasizing general patterns rather than detailed percentages, in order to improve clarity and readability.

Lines 12-35.

**2. The terms “CO₂ injection regimes,” “CO₂ injection times,” and “CO₂ injection” are used interchangeably. Terminology should be standardized throughout the manuscript.**

Terminology has been standardized throughout the abstract and manuscript, and the term “CO₂ injection times” is now used consistently.

Line 28.

**3. The abstract suggests the investigation of synergistic effects; however, the results clearly indicate a lack of additive effects. This nuance should be stated more explicitly and with a less optimistic tone.**

The abstract has been revised to explicitly state that combined treatments did not produce additive or synergistic effects, and the tone has been adjusted to reflect a more cautious interpretation of the results.

Line 30.

**Introduction**

**1. There is repetition of ideas. The relevance of microalgae and growth optimization is reiterated across multiple paragraphs with limited conceptual progression. For example, lines 37–45 and 47–49 essentially address the same argument.**

We have revised the Introduction to eliminate repetitive statements regarding the relevance of microalgae and biomass optimization. The section has been reorganized to improve conceptual progression, moving from the ecological and biotechnological importance of microalgae to the necessity of optimizing growth for scalable applications, followed by current knowledge gaps.

Lines 42-51.

**2. The final objective of the study (lines 65–70) refers to both growth and nutritional quality, which creates expectations of more in-depth nutritional analyses than those performed. The objective should be clarified, emphasizing that growth is the main focus and that nutritional analysis is exploratory or complementary.**

The study objective has been revised to emphasize that growth performance and biomass enhancement constitute the primary focus of the work. Nutritional parameters are now explicitly described as complementary indicators used to contextualize growth responses, rather than as a central analytical objective. This adjustment better aligns the stated objective with the scope of the results presented.

Lines 95-102.

**Materials and Methods**

**1. This section contains excessive operational detail.**

The Materials and Methods section has been revised to reduce excessive operational detail. Non-essential procedural descriptions have been condensed while retaining all information necessary for reproducibility.

**2. The description of culture isolation and cleaning procedures is too long for a research article.**

The description of culture isolation and cleaning procedures has been substantially condensed to include only key steps required for understanding and reproducibility.

Lines 113-119.

**3. It is recommended to condense operational details, retaining only what is essential for reproducibility.**

Operational descriptions have been streamlined throughout the section. Only essential experimental conditions, culture parameters, and analytical procedures have been retained.

**4. It should be clearly stated that species identification was based on morphology only, without molecular confirmation.**

The manuscript has been revised to explicitly state that species identification was based exclusively on morphological characteristics following taxonomic keys and Algaebase references, without molecular confirmation.

Lines 130-133.

**5. The rationale for selecting the concentration ranges of bioactive substances is not clearly explained.**

A clarification has been added to section 2.3 explaining that the selected concentration ranges were defined based on preliminary exploratory assays and literature reports describing biological activity at low concentrations in microalgal systems.

Lines 186-196.

**6. Some substances show positive effects only at very specific concentrations, but this is not discussed methodologically.**

The methodology has been revised to clarify that the selected concentration ranges were intended to identify potential stimulatory windows, acknowledging that positive effects may occur only at specific concentrations.

Lines 186-196.

**7. References should be included to justify the selected concentrations, or the authors should explicitly state that these were exploratory concentrations.**

The manuscript now specifies that the tested concentrations were exploratory and informed by literature describing biological activity ranges of similar compounds in microalgal systems.

Lines 186-196.

**8. Terminology should be standardized, for example: “Indol-3-butíric acid” vs. “Indole-3-butyric acid”; “naphtalenacético” vs. “naphthaleneacetic”.**

Terminology has been standardized throughout the manuscript. All compound names have been revised to ensure consistent English nomenclature.

Line 198 (Table 1).

**9. Light intensity is described as “estimated” (80–150 μmol m⁻² s⁻¹). Is there a limitation preventing direct measurement of this variable? If intensity was not controlled, conclusions based solely on spectral effects should be interpreted cautiously.**

In the revised manuscript, light intensity was directly measured at the culture surface using a lux meter. The recorded values were approximately 170 lux (red), 144 lux (blue), 273 lux (green), 280 lux (yellow), and 283 lux (white control). We acknowledge that lux measurements do not directly correspond to photosynthetically active radiation (PAR); however, identical LED configurations and reflective chamber geometry were maintained across treatments to ensure comparable illumination conditions. The manuscript has been updated in Section 2.4 to report the measured values and clarify the basis for spectral comparisons. Interpretations remain focused primarily on spectral distribution under controlled experimental settings.

Lines 217-224.

Lines 669-671.

**10. Why is biomass recovery from the culture medium described only in Section 2.5?**

We appreciate the reviewer’s observation. Biomass recovery was described in Section 2.5 because dry weight determination was required specifically for CO₂ fixation calculations. Routine growth assessment throughout the study was performed using cell density measurements obtained by spectrophotometry and cell counting (Section 2.6), and therefore biomass harvesting was not necessary for those analyses. To improve clarity, we have added a statement in Section 2.6 explaining that dry biomass determination was conducted exclusively for CO₂ fixation analysis.

Lines 245-248.

Lines 295-297.

**11. Was the same wavelength (630 nm) used for biomass determination for all species? This wavelength may not be optimal for all microalgae analyzed.**

Optical density at 630 nm was selected because it is commonly used for estimating microalgal biomass and provides reliable correlations with cell density while minimizing interference from chlorophyll absorption peaks. Importantly, species-specific calibration curves were constructed for each microalga by correlating OD₆₃₀ with direct cell counts. This approach ensured accurate biomass estimation for each species independently, regardless of potential differences in spectral absorption characteristics. We have clarified this justification in Section 2.6 and included additional supporting references.

Lines 252-255.

**12. Is there a bibliographic reference supporting the method used to construct the calibration curves? Were the calibration curves established after 15 days of growth monitoring?**

The calibration curves were constructed by correlating OD₆₃₀ measurements with direct cell counts over a 30-day monitoring period, covering different growth phases. Measurements were performed every 24 h during the first 15 days to accurately characterize the exponential phase, and every 48 h thereafter. The calibration equations were derived from linear regression analyses between optical density and cell density. This approach is consistent with previously reported spectrophotometric biomass estimation methods in microalgae. We have clarified this description in Section 2.6 and included additional supporting references.

Lines 252-259 and 266-271.

**13. Why were only one initial point and one final point (12 days) used for the main tests?**

The 12-day experimental period was selected based on prior exploratory assays conducted under similar culture conditions and is consistent with previous studies from our research group evaluating microalgal growth responses over 10-day periods under comparable setups [1,2]. These studies indicate that treatment effects are typically most evident during the late exponential phase (approximately days 10–15). Additionally, extended 30-day assays (included in the Supplementary Material) confirmed that maximal differences among treatments occurred within this time window, while later measurements reflected stationary-phase stabilization. We have clarified this rationale in the revised manuscript.

1. Herrera N, Echeverri F. Evidence of Quorum Sensing in Cyanobacteria by Homoserine Lactones: The Origin of Blooms. Water. 2021; 13:1831. https://doi.org/10.3390/w13131831

2. Herrera N, Llanes A, Echeverri F. Effect of pesticides and tributaries on the growth and development of Microcystis aeruginosa and Cylindrospermopsis raciborskii. Algal Research. 2025; 85:103876. https://doi.org/10.1016/j.algal.2024.103876

Lines 153-160.

**14. Regarding statistical analysis, why was a more conservative post hoc test (e.g., Tukey) not applied?**

Fisher’s LSD test was applied only after verification of model assumptions and a significant ANOVA for each independent linear model (bioactive substances, wavelengths, CO₂ treatments, and combined conditions) and species. Although the bioactive substances factor involved multiple treatments, analyses were conducted within each species and factor separately using a balanced design with triplicates. LSD was selected to maintain statistical power for detecting biologically meaningful differences relative to the control. Furthermore, the magnitude of the observed effects and the highly significant p-values (S3 File. Supplementary data tables) indicate robust treatment differences. The statistical analysis section has been clarified accordingly.

Lines 357-375.

**Results**

**1. The resolution of Figures 2–9 should be improved.**

All figures have been replaced with high-resolution versions to improve clarity and readability.

**2. Many figures contain excessive visual information (numerous LSD test letters), making interpretation tiring. Simplification of the graphical presentation is recommended, if possible.**

The graphical presentation has been simplified to improve readability. The number of statistical letters displayed has been reduced where possible, and emphasis has been placed on the most relevant treatment comparisons.

**3. Growth curves are not fully explored in the text, representing a missed opportunity to enrich the dynamic interpretation of growth.**

A brief description of the growth dynamics has been incorporated into the Results section to highlight differences in lag phase duration, exponential growth behavior, and stabilization patterns observed among treatments.

Lines 396-399.

Lines 438-440.

Lines 467-469.

Lines 504-505.

**4. There is excessive repetition of numerical values, p-values, and LSD letters in the text. Reducing numerical detail and emphasizing general trends is suggested.**

The Results section has been revised to reduce excessive numerical repetition.

**5. The Results section contains limited interpretative synthesis, with extensive description and little integration across findings. Better integration of results is recommended.**

The Results section has been revised to improve integration across species and experimental factors. Comparative trends (e.g., common positive effects of *Aloe vera* or blue/red light across species) are now briefly highlighted to provide a more cohesive presentation of the findings while maintaining separation from the Discussion section.

**6. Information presented in figures and tables is extensively repeated in the text. Reorganization is suggested to avoid redundancy.**

We agree and have reorganized the Results section to avoid redundancy.

**7. It should be clearly stated that combined treatments are not necessarily advantageous and may induce physiological stress.**

The Results section now clearly states that combined treatments were not consistently advantageous and, in certain cases, resulted in lower growth responses compared to individual treatments, suggesting potential physiological stress effects.

Lines 406-409.

Lines 442-444.

Lines 480-482.

Lines 517-519.

**8. Why is no statistical analysis presented for the responses shown in Table 2?**

The compositional analysis presented in Table 2 was conducted on biomass obtained under the best-performing condition for each species. Due to the nature of the analytical procedure, measurements represent single determinations per condition and are therefore presented as descriptive data. This has been clarified in the revised manuscript.

Lines 539-544.

**9. The term “significant nutritional improvement” should be avoided unless supported by appropriate statistical analysis, which should be included if available.**

The term “nutritional improvement” has been removed and replaced with descriptive language.

Line 765-766.

**Discussion**

**1. The Discussion includes repeated ideas, long paragraphs, and occasionally redundant language, which compromises fluency and objectivity. Revision is recommended to remove repetition and synthesize arguments.**

The Discussion section has been revised to improve clarity and conciseness. Repeated ideas have been removed, lengthy paragraphs have been divided, and arguments have been synthesized to enhance fluency and objectivity.

**2. In some passages, results are presented as robust evidence of physiological mechanisms, whereas the data are essentially observational. Such statements should be revised. For example, direct attribution of effects to cytokinins, vitamins, or antioxidants is made without direct biochemical measurements. More cautious language is recommended (e.g., “may be associated with,” “possibly related to”).**

Statements suggesting direct physiological mechanisms have been revised to reflect the observational nature of the data. Mechanistic interpretations are now presented using cautious language (e.g., “may be associated with,” “possibly related to”) to avoid overinterpretation.

Lines 570-573.

**3. The relevance of Aloe vera and coconut water as sustainable alternatives is reiterated several times using similar arguments.**

The argument regarding the sustainability and accessibility of Aloe vera and coconut water has been streamlined to avoid redundancy while maintaining its relevance.

**4. Light intensity was not rigorously controlled, only estimated, and CO₂ injection was based on time rather than dissolved concentration. These limitations should be more explicitly acknowledged to avoid overinterpretation of the results.**

The limitations regarding light intensity estimation and the use of injection time rather than dissolved CO₂ concentration have now been explicitly acknowledged in the Discussion. These factors are discussed as potential sources of variability, and interpretations have been adjusted accordingly.

Lines 669-671.

Lines 692-700.

**5. The discussion of combined factors (bioactives + light + CO₂) could be more concise and** **more directly connected to the data. The central argument (physiological interference and** **non-linear responses) becomes diluted in lengthy paragraphs.**

The section discussing combined factors has been revised to be more concise and directly linked to the observed PGI responses. The argument regarding potential physiological interference and non-linear interactions has been clarified and streamlined.

Lines 719-720.

**Conclusions**

**1. The conclusions refer to “nutritional quality,” but the data show modest and inconsistent changes. Reducing the emphasis on this aspect or explicitly characterizing it as exploratory is recommended.**

The Conclusions section has been revised to reduce the emphasis on nutritional quality. Changes in biomass composition were modest and inconsistent across species; therefore, compositional outcomes are now presented as exploratory observations rather than definitive improvements.

Lines 756-769.

**2. The conclusions could more strongly emphasize that the results are specific to the experimental conditions adopted in this study.**

The Conclusions have been modified to explicitly state that the results are specific to the experimental conditions adopted in this study, including estimated light intensity and CO₂ injection based on time rather than dissolved concentration, to avoid overgeneralization.

Lines 756-769.

**Reviewer #2:**

The manuscript entitled “Enhancing microalgal productivity through bioactive substances, light, and CO₂”. This manuscript need revision before publication.

**1. Line 86: and remove unwanted particles. What are the unwanted martials and how authors remove them in microalgae culture?**

The reference to “unwanted particles” corresponded to coarse soil debris present during the initial enrichment step. To clarify this, the manuscript has been revised. The previous wording suggesting removal through centrifugation has been removed to avoid confusion.

**2. Line 86-90 are confusing need to improve it.**

In response, the section has been rewritten for clarity and logical sequence. Additionally, following Reviewer #1’s recommendation to condense the description of isolation and cleaning procedures, the methodology was simplified in the main text to include only essential steps required for understanding and reproducibility.

Lines 113-119.

**3. Lin 93 what was the concentration of microalgae (OD) in the start of the experiment.**

Initial optical density (OD) values were measured at the beginning of each assay. Although OD was not standardized across independent experiments, treatments and their corresponding controls within each experimental set were inoculated from the same actively growing pre-culture to ensure comparable starting biomass. Initial OD values at day 0 were approximately 0.025 for *A. platensis*, 0.018 for *C. vulgaris*, 0.013 for *A. falcatus*, and 0.012 for *T. dimorphus*. This information has now been clarified in the Methods section 2.2.

Lines 148-151.

**4. Why authors use such low light intensity (100 μmol m⁻² s⁻¹,) for low 12 h:12 h light/dark photoperiod. The optimized is 18:6.**

The light intensity (100 μmol m⁻² s⁻¹) and 12:12 h photoperiod were selected as standard, moderate cultivation conditions to maintain physiological stability across species and avoid potential photoinhibition or stress responses associated with prolonged illumination. The objective of this study was not to optimize photoperiod or irradiance, but rather to evaluate the effects of bioactive substances, spectral quality, and CO₂ supplementation under controlled baseline conditions. A 12:12 regime is widely reported in laboratory-scale microalgal cultivation and allows adequate metabolic recovery during the dark phase.

**5. How authors determine the growth of microalgae.**

Microalgal growth was determined by cell counting and optical density measurements, as described in Section 2.6. To improve clarity, a brief reference to the growth determination methodology has now been added in Section 2.2 to guide the reader.

Lines 159-160.

**6. Table 1. why authors choose such costly bioactive compound rather than any other economic material for imrove the growth of microalgae. Is this study is economic feasible.**

The selected bioactive compounds included both commercially available synthetic regulators and natural extracts in order to compare their relative growth-promoting potential under controlled experimental conditions. While some synthetic compounds may represent higher-cost inputs, their inclusion allowed benchmarking against substances commonly reported in the literature.

We acknowledge that a formal economic feasibility analysis was not conducted, as this study focused on biological performance rather than techno-economic evaluation. To avoid overstatement, the term “low-cost” has been revised throughout the manuscript and replaced with “naturally derived,” and a statement has been added indicating that future studies should assess economic feasibility and scalability under industrial conditions.

Lines 84-85 and line 596.

**7. Where is the growth curve. Provide it in supplementary material.**

The calibration (growth) curves relating OD₆₃₀ to cell density for each species have now been included in the Supplementary Material (S4 File).

Lines 269-271.

**8. Authors may add the flowing publication in discussion to improve the quality of paper 10.1016/j.pbi.2025.102696, 10.1016/j.bcab.2024.103315, 10.3390/su16167075**

The recommended publications have been carefully reviewed and incorporated into the Discussion section where relevant, particularly in relation to recent advances in microalgal biostimulation, sustainability considerations, and integrated cultivation strategies. These additions strengthen the contextualization of our findings within current literature.

1. as well as differential regulation of carbon storage and photoacclimation processes under interacting light and carbon conditions [50].

Lines 686-687. **10.1016/j.pbi.2025.102696**

2. in biomass under defined CO₂ pulses confirm that carbon availability can directly influence productivity, and comparable species-dependent optimization thresholds have been described under controlled flue gas atmospheres with varying CO₂ concentrations [51].

Lines 687-690. **10.3390/su16167075**

3. Similar improvements under red light have been reported in other microalgae, where spectral modulation enhanced biomass accumulation and metabolite production under defined stress conditions [46].

Lines 660-662. **10.1016/j.bcab.2024.103315**

**9. Figures are not in good resolution. Provide the figure with good resolution.**

All figures have been re-exported at high resolution in accordance with the journal guidelines.

**Reviewer #3:**

This article addresses an interesting and important topic: the search for conditions that enhance the productivity of microalgae, which in turn are a promising resource for the production of valuable products, biofuels, and other materials. The article presents a methodology and applies an interesting approach that allows to determine the influence of individual growth factors (stimuli) and to study the complex (simultaneous) impact of several factors. The following comments need to be made:

**1. The authors should note that the introduction, specifically the analysis of previously conducted studies, is extremely brief. It is important to present not only the general results of such studies but also a brief description of the methods used. It would be desirable to include in the introduction the results of the combined effects of various growth factors on microalgal biomass, if such studies have been conducted previously.**

The Introduction has been substantially expanded to provide a more detailed analysis of previously conducted studies, including not only general findings but also brief descriptions of the methodological approaches employed. In particular, we have incorporated literature addressing the combined effects of multiple cultivation factors on microalgal biomass production.

Specifically, we now reference studies evaluating the synergistic interaction between light intensity and dissolved inorganic carbon, multifactor kinetic modeling approaches integrating light, carbon, and nutrient availability, and dual-regime cultivation strategies such as mixotrophy. These additions contextualize our multifactor experimental design and clarify how the present study extends previous work by evaluating bioactive substances, spectral quality, and CO₂ supplementation both individually and in combination across four taxonomically distinct species. We believe these revisions strengthen the theoretical framework of the manuscript and more clearly position our contribution within current research trends.

Lines 70-81.

**2. Given the low solubility of CO2, it is unclear what processes could have achieved any noticeable changes in microalgal biomass productivity with such short exposure times (30 to 120 s). Experiments assessing the effect of CO2 on microalgal growth are typically conducted with continuous bubbling of the culture fluid with a mixture of CO2 and air with an elevated carbon dioxide concentration. Can the authors explain why this particular mode of CO2 addition to the culture fluid was chosen for the experiments?**

Continuous bubbling with air–CO₂ mixtures is indeed a common approach in large-scale or optimization studies. However, in the present work, CO₂ was supplied as short, controlled daily pulses rather than continuous enrichment. This experimental design was selected to evaluate the physiological response of microalgae to transient increases in dissolved inorganic carbon under otherwise constant aeration conditions (1.0 L/min air supply). Microalgae possess carbon concentrating mechanisms (CCMs) that enable efficient uptake and assimilation of inorganic carbon even under fluctuating availability. Short CO₂ injections can temporarily elevate dissolved CO₂ and shift carbonate equilibrium toward increased bicarbonate availability, which may be rapidly assimilated.

Our objective was not to maximize carbon saturation but to compare relative species-specific responses to defined carbon input regimes within a controlled framework. We have clarified this rationale in Section 2.5 and further discussed it in the Discussion section to better contextualize the experimental design.

Lines 239-243.

Lines 692-700.

**3. The authors should clarify the composition of the gases during aeration during the main part of the experiments – was it air with a standard CO2 concentration? In section 2.2, the authors write: “Aeration was continuous and maintained at 1.0 L/min.”**

Continuous aeration during culture maintenance (Section 2.2) was performed using filtered ambient air supplied at 1.0 L/min. No enriched gas mixture was used during routine aeration.

Line 142.

Elevated CO₂ (99.95% purity) was applied only during the specific treatments described in Section 2.5 as controlled daily pulse injections. We have clarified this distinction in the revised manuscript to avoid confusion between baseline aeration and experimental CO₂ supplementation.

Line 233.

**4. Figures labeled "A" are too information-heavy and difficult to understand and analyze. Perhaps a more detailed analysis should be provided in Table B in File S3?**

In response, the figures have been simplified to improve clarity and readability, highlighting the most relevant comparative trends. The detailed numerical data underlying these figures are provided in Table B (File S3), which contains the complete PGI values and biomass composition results for all treatments and species. The Discussion section is based directly on these quantitative results. To improve transparency, we have now explicitly referred to Table B in S3 within the Discussion to guide readers to the full dataset for detailed examination.

Line 593.
